# Exposure-response relationship between transportation noise and cardiovascular disease outcomes: a systematic review and meta-regression analysis

**DOI:** 10.1038/s41370-026-00856-9

**Published:** 2026-04-06

**Authors:** Elia Gonzato, Tessa M. I. Haverkate, Susanne Breitner-Busch, Anette Kocbach Bølling, Gunn Marit Aasvang, Michael Brauer, Vanessa Garcia, Sandra Spearman, Brecht Devleesschauwer, Juanita A. Haagsma, Periklis Charalampous

**Affiliations:** 1 Department of Public Health, Erasmus MC University Medical Center Rotterdam, Rotterdam, The Netherlands; 2 Institute for Medical Information Processing, Biometry, and Epidemiology, Faculty of Medicine, LMU, Munich, Germany; 3 Institute of Epidemiology, Helmholtz Zentrum München-German Research Center for Environmental Health (GmbH), Neuherberg, Germany; 4 Department of Air Quality and Noise, Norwegian Institute of Public Health, Oslo, Norway; 5 Centre for Disease Burden, Norwegian Institute of Public Health, Bergen, Norway; 6 School of Population and Public Health, University of British Columbia, Vancouver, BC Canada; 7 Institute for Health Metrics and Evaluation, University of Washington, Seattle, WA USA; 8 Department of Epidemiology and Public Health, Sciensano, Brussels, Belgium; 9 Department of Translational Physiology, Infectiology and Public Health, Ghent University, Merelbeke, Belgium; 10 Institute of Health and Society (IRSS), Université catholique de Louvain, Brussels, Belgium

**Keywords:** Transportation noise, noise pollution, cardiovascular disease, burden of disease, exposure-response functions, Burden of Proof

## Abstract

**Background:**

Current risk assessments of cardiovascular disease (CVD) outcomes attributable to transportation noise rely on estimates from the 2018 WHO Environmental Noise Guidelines. Since the publication of these guidelines, several studies have been conducted to determine the association between transportation noise sources and CVD; however, recent meta-analyses have not derived updated exposure-response functions.

**Objective:**

We reviewed epidemiological evidence linking long-term exposure to road traffic, railway, and aircraft noise with non-fatal and fatal myocardial infarction, ischemic heart disease, stroke, and ischemic stroke, and derived exposure-response functions using the conventional and Burden of Proof (BoP) methodologies.

**Methods:**

We systematically searched databases for cohort or case-control studies that determined the associations between non-fatal and/or fatal myocardial infarction, ischemic heart disease, stroke, and ischemic stroke and long-term exposure to road traffic, railway, aircraft noise in general populations. Exposure-response functions were generated using the conventional natural cubic splines and Burden of Proof Risk Function approaches.

**Results:**

Twenty-six studies met our eligibility criteria. Road traffic noise was associated with 1% increase in the combined risk of stroke incidence and mortality (RR = 1.01, 95%CI: 1.00–1.02, *p*-value = 0.04), and with 5% increase under the BoP framework (RR = 1.05, 95%UI: 1.03–1.07). Railway noise was associated with 1% increase in myocardial infarction outcomes (RR = 1.01, 95%CI: 1.01–1.01, *p*-value < 0.0001), and with 16% increase under the BoP framework (RR = 1.16 95%UI: 1.07–1.26). Of the twelve risk-outcome pairs examined, five showed no evidence of association, four showed weak evidence, and the remainder lacked credible evidence or did not meet the BoP criteria. Compared with the natural splines approach, the BoP framework produced more plausible exposure-response curves.

**Significance:**

This study adds to the existing literature by providing a comprehensive comparison of the association between long-term exposure to transportation noise sources and CVD outcomes using both conventional and BoP methodologies.

**Impact statement:**

This is the first study to apply the conventional meta-regression and Burden of Proof methodologies to systematically quantify and evaluate associations between long-term exposure to transportation noise sources (i.e., road traffic, railway, and aircraft) and combined risk of fatal and non-fatal cardiovascular disease outcomes, including myocardial infarction, ischemic heart disease, stroke, and ischemic stroke. The application of these two approaches to deriving exposure-response functions provides additional insights into the quantification of the burden of disease attributable to transportation noise. Our findings using the Burden of Proof framework on transportation noise and CVD outcomes advance the integration of an additional environmental risk factor and propose new risk-outcome pairs for potential inclusion in the Global Burden of Disease study.

## Introduction

Transportation noise has emerged as a major environmental stressor that not only increases the risk of sleep disturbance and noise annoyance but also contributes to cardiovascular diseases (CVD), such as ischemic heart disease (IHD) and stroke, as well as CVD-related risk factors, such as hypertension and obesity [1–4]. To quantify the impact of transportation noise on population health, an exposure-response function between specific transportation noise sources—such as road traffic, railway, and aircraft noise—and associated health outcomes is warranted.

The 2018 Environmental Noise Guidelines for the WHO European Region were developed by an expert panel, employing the grading of recommendations, assessment, development, and evaluation (GRADE) tool to evaluate the quality of evidence from studies examining associations between various noise sources and health outcomes, and to derive noise source-specific exposure-response functions [5]. In these guidelines, the expert panel concluded that the quality of evidence supporting the association between transportation noise and CVD outcomes varied considerably. High-quality evidence was found for the association between road traffic noise and IHD. In contrast, the evidence for the association with railway noise was considered low-quality, and that for aircraft noise was deemed very low. The expert panel also concluded that the quality of evidence linking road traffic noise to stroke was of moderate quality. However, the data collection to support the above-described conclusions covered the period from January 2000 to August 2015 [5]. Since then, several population-based studies have been conducted to determine the association between transportation noise sources and CVD outcomes, yielding different results, likely due to heterogeneity in exposure assessment methods and/or population heterogeneity in demographic characteristics and contextual factors [6–10].

Exposure-response functions linking transportation noise with health outcomes are traditionally established through quantitative synthesis of studies, often involving traditional meta-analyses. These analyses combine effect sizes extracted from epidemiological studies and weight them by the inverse of their variances. Between-study heterogeneity is quantified, and mixed models that account for variability between studies are employed to adjust the weighting of each estimate in the pooled analysis [11, 12]. A more recent approach to deriving exposure-response functions is the Meta-Regression-Bayesian, Regularized, Trimmed (MR-BRT), which is part of the Burden of Proof (BoP) framework [13, 14]. The MR-BRT approach facilitates the detection and removal of outliers through a standardized trimming methodology and enables the integration of heterogeneous data into one model, thereby ensuring stable estimations of exposure-response curves [13, 15, 16]. This framework also accounts for heterogeneity in effect estimates between studies that is not explained by study-level factors, such as exposure measurement error or confounding. In addition, the BoP framework includes a risk-outcome score (ROS), which quantifies the strength of evidence based upon both the magnitude of the mean effect estimate and the degree of unexplained between-study heterogeneity [16]. The application of these two approaches to deriving exposure-response functions provides additional insights into quantifying the burden of disease attributable to transportation noise.

Therefore, we aimed to conduct a systematic review that includes a qualitative synthesis and quantitative meta-analysis of existing epidemiological evidence on the association between long-term exposure to transportation noise sources (i.e., road traffic, railway, and aircraft) and CVD outcomes. These outcomes included non-fatal (measured by incidence) and fatal (measured by mortality) myocardial infarction, IHD, stroke, and ischemic stroke. We also aimed to derive and critically discuss exposure-response functions using both the conventional meta-regression and BoP approaches to obtain conservative estimates and ratings of evidence strength.

## Methods

### Search strategy and selection criteria

We systematically searched the literature across six bibliographic databases (PubMed, Embase, Web of Science, Cochrane Central, Scopus, and Global Index Medicus) and an additional search engine (Google Scholar) from inception to July 4, 2023. We also hand-searched the reference lists of all identified systematic reviews and meta-analyses to find additional eligible records. The search strategy, developed by an experienced librarian of the Erasmus MC, is available in the Supplementary material (pp 4). The protocol has been registered in the PROSPERO database (CRD42023444656).

Without any geographical restrictions, we considered published papers if they met the following criteria: (i) cohort or case-control studies that determined the association between non-fatal and/or fatal major CVD outcomes, and long-term transportation noise source (i.e., road traffic, railway, aircraft) in general populations; (ii) studies that quantified a relative measure of association or number of cases and non-cases among exposed groups *versus* non-exposed comparators; (iii) studies that quantified the association for a certain increase in noise exposure (dB), either categorically or by assuming linearity (i.e. the risk in increase remains constant per noise exposure interval), and accounted for uncertainty; (iv) studies that explicitly reported on the noise exposure levels (e.g., 24 h average sound levels weighted with a penalty of 5 dB for evening time noise and 10 dB for nighttime noise; day-evening-night noise level (L_den_)) and defined how and when the studied exposure was modeled; (v) studies that explicitly defined the studied disease outcomes based on the International Classification of Diseases codes; and (vi) studies published in English. It should be noted that if multiple publications reported on the same population, we considered the one with the longest follow-up duration, and consequently, the greatest number of disease cases. Details on the case definitions and exposure assessment criteria considered can be found in the Supplementary material (pp 5). We excluded cross-sectional, ecological, animal, and case studies, as well as publications, such as editorials, letters-to-the-editor, commentaries, and conference proceedings that lacked essential information (e.g., effect sizes and/or appropriate uncertainty information).

### Data screening and extraction

After removing duplicates, we applied the above criteria to screen the titles and abstracts of all identified records, as well as the full texts of potentially eligible records. Two reviewers (PC & JH) independently conducted title/abstract screening using the EndNote 21 software. One researcher (PC) manually searched the reference lists of all identified systematic reviews and meta-analyses in order to detect additional studies. PC & EG documented all eligible records in an Excel spreadsheet, shared it with an expert in the field (GMA), and discussed the main reasons for inclusion/exclusion. PC, EG, JH & GMA discussed any disagreements arising from the eligibility criteria.

In addition, PC & EG used a validated data extraction form developed by the Global Burden of Disease (GBD) researchers to extract data pertaining to study characteristics, disease outcome, sources of noise exposure, adjusted confounders, and effect sizes and their uncertainty (Supplementary material pp 7). Where available, we extracted effect size estimates from models adjusted for factors including age, sex, socio-economic status, and correlated exposures, such as particulate matter smaller than 2.5 μm in aerodynamic diameter (PM_2.5_) and nitrogen dioxide (NO_2_). When multiple estimates were available, we prioritized those derived from the most adjusted models (i.e., models that accounted for air pollutants) over those that did not. In cases where transportation noise exposure was measured both as L_den_ and L_night_ (i.e., equivalent continuous sound pressure level when the reference time interval is the night), the former was prioritized due to its overlap with the latter. PC, JH, GMA, and EG discussed any disagreements arising from data extraction items. It should be noted that researchers engaged in every stage of the review process received training from GBD researchers.

### Methodological quality

Two reviewers (PC & EG) independently evaluated the methodological quality of the included papers using the Newcastle-Ottawa Scale (NOS) [17]. The NOS utilizes a nine-point scale, assigning points across three main domains: selection of participants (four points), comparability of cohorts or case-control groups (two points), and assessment of outcomes and adequacy of follow-up (three points). We classified studies into three categories based on their methodological quality: low (score: 0–3 points), medium (score: 4–6 points), and high (score: 7–9 points). Any disagreements were resolved by consensus.

### Data analysis and synthesis

#### Systematic review

A narrative synthesis was conducted by classifying studies according to their study characteristics (e.g., year of publication, geographical location, methodological quality, model adjustments, risk-outcome pairs assessed, and CVD outcomes covered) and whether they examined non-fatal or fatal events.

#### Data pre-processing

Records were deemed appropriate for meta-analysis if at least three independent studies had an equivalent exposure, disease outcome, and health indicators, and if they reported the same or equivalent effect statistics, thereby enabling meaningful and interpretable pooling of estimates. Risk measures were reported in varying formats (i.e., categorical or continuous values). To ensure consistency, we standardized these measures by converting categorical data to a common 10 dB increment and scaling the midpoints to the L_den_ indicator [18]. These standardized values were modeled against the associated risk measure using the *dosresmeta* package [19]. A detailed description of the conversion process is available in the Supplementary material (pp 15).

#### Conventional meta-analyses and meta-regression

Fixed-effects models were fitted, and between-study heterogeneity was quantified with Cochran’s Q and I [2] statistics (Supplementary material pp 16). If the *p*-value associated with the heterogeneity test was lower than 0.05, a random-effects model was preferred. Publication and reporting bias were assessed with visual inspection of funnel plots. Relative risk (RR) estimates, 95% confidence intervals (95%CI), and I [2] were reported. In an additional step, a meta-regression was employed to model log(RR) against noise exposure levels using natural cubic splines [20]. To characterize the shape of the relationship between exposure and the log(RR), splines with three knots were used to account for potential non-linear trends across the noise exposure range. Exposure-response functions were plotted from meta-regression results. It should be noted that studies trimmed from the MR-BRT approach were excluded from the conventional meta-analyses and meta-regression, facilitating comparisons between the two approaches. Supplemental conventional meta-analyses on major fatal and fatal CVD outcomes, without pooling trimmed estimates, were conducted (Supplementary material pp 20). All statistical analyses were performed using R version 4.3.2 [21].

#### Burden of proof (BoP) framework

We applied the GRADE guidelines to create a series of binary covariates capturing potential sources of bias [22]. Across all risk-outcome pairs analyzed, the covariates primarily included those measuring the representativeness of the study sample; whether exposure was measured at the overall population or a subpopulation only; risk of selection bias; reverse causation; and estimates uncontrolled for major confounders (Supplementary material pp 14). The potential effect of bias covariates was tested using the MR–BRT automated covariate selection process, which applies a LASSO strategy to identify statistically significant covariates at a threshold of 0.05 [16, 23]. To quantify between-study heterogeneity, the framework accounts for within-study correlations, overall study variability, and the influence of a limited number of studies. The Fisher information matrix, along with the final uncertainty estimate, was then generated to quantify the posterior uncertainty associated with the fixed effect (as in the conventional meta-analytical approach) [13]. RR estimates, with and without adjustment for between-study heterogeneity, were presented with corresponding 95% uncertainty intervals (95%UI). Publication and reporting bias were assessed with visual inspection of funnel plots (as in the conventional meta-analytical approach). To characterize the shape of each exposure-risk association, knots were placed anywhere within the 5th and 95th percentiles of the data; shape constraints were applied to ensure that the resulting exposure-response functions were concave down and exhibited monotonic behavior. We estimated the Burden of Proof Risk Function (BPRF), reflecting the most conservative estimate of the association between transportation noise sources and CVD. ROS were calculated from the BPRF as the log(BPRF) divided by 2. ROS were categorized into star-rating categories ranging from one to five; a one-star (ROS value: ≤0.0) indicates weak evidence of association, while a five-star (ROS value: >0.62) indicates very strong evidence of association [13, 16]. It should be noted that risk-outcome pairs with a zero-star association do not meet the GBD inclusion criteria and were thus excluded from further discussion. Further details of the BoP framework have been described elsewhere[16]. All statistical analyses were performed using R version 4.3.2 and Python version 3.10.4 [21, 24].

### Research and reporting practices

This study complies with both the Preferred Reporting Items for Systematic Reviews and Meta-Analyses (PRISMA; Supplementary material pp 24) and the GATHER (Supplementary material pp 27) guidelines [25, 26]. All code used for both the conventional meta-regression (https://github.com/egonzato/noisecvds_meta-analysis) and MR-BRT analyses (https://github.com/ihmeuw-msca/burden-of-proof/) are publicly available.

## Results

### Literature review

**Fig. 1:**
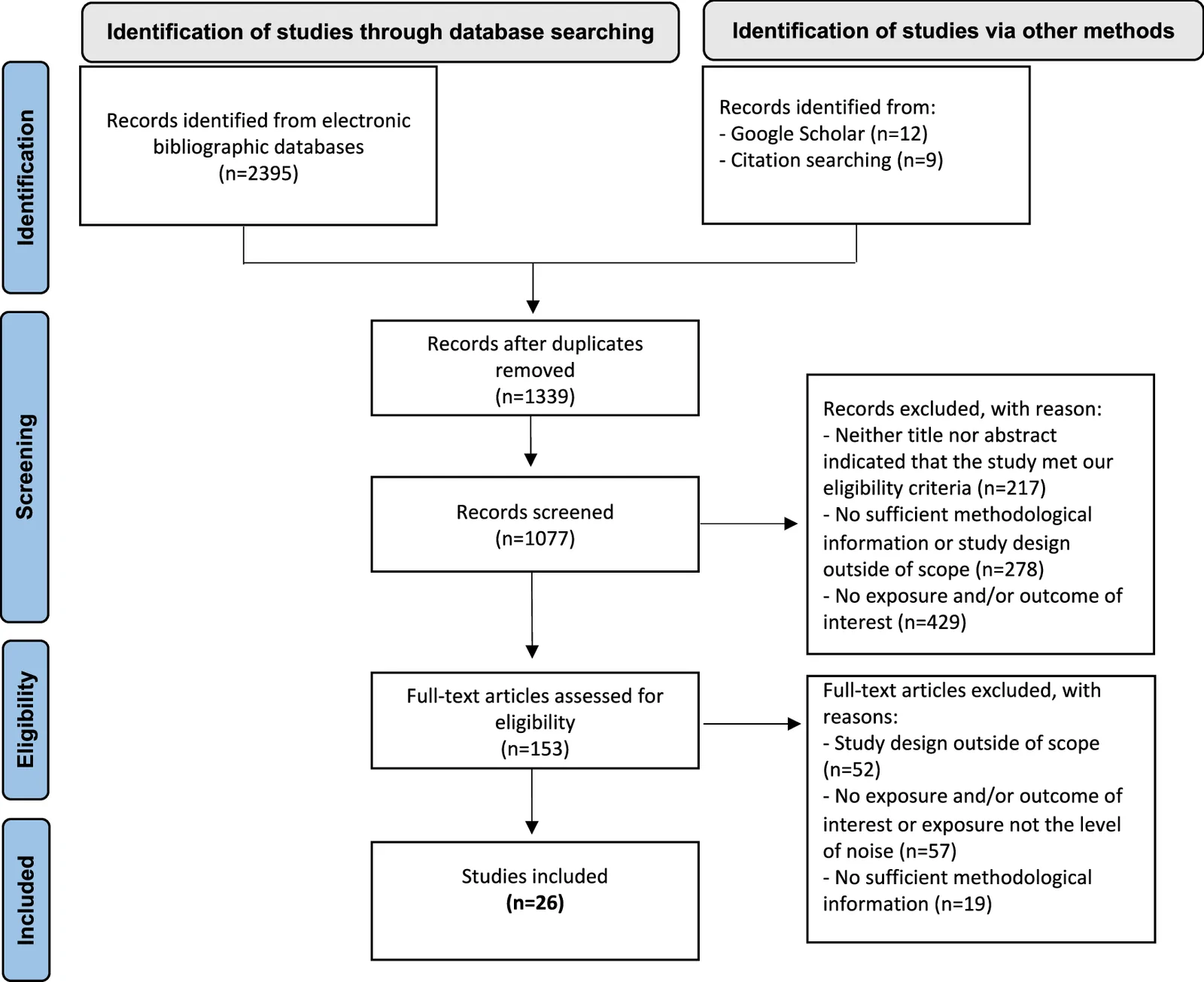
PRISMA flowchart. Flowchart of the literature search and study selection.

After removing duplicates, we identified a total of 1339 relevant records through electronic database searches, gray literature, and handsearching. The full texts of 153 articles were systematically reviewed and led to the final inclusion of 26 unique studies (Fig. 1).

### Study characteristics and methodological quality assessment

Of the 26 studies considered eligible, the vast majority (*n* = 25) were conducted at a single-country level, with 23 performed in European countries (Table 1 & Supplementary Fig. S1, Supplementary material pp 19) [6, 8, 27–46]. The lowest number of studies was conducted in the United States (*n* = 1) [47] and Canada (*n* = 2) [48, 49], whereas the highest number of studies was conducted in Denmark (*n* = 8) [30, 33, 35, 36, 40–43], followed by Germany (*n* = 5)[27, 37, 38, 44, 46]. The risk-outcome pair that was studied the most was road traffic noise and myocardial infarction (*n* = 16) [6, 27–29, 31–34, 36, 38, 41, 43–45, 48, 49], followed by road traffic noise and stroke (*n* = 11)[6–8, 29–32, 34, 35, 37, 39, 40]. The risk-outcome pairs involving railway (*n* = 3) and aircraft noise (*n* = 4) were the least studied (Table 1 & Supplementary Fig. S2, Supplementary material pp 19). Regarding model adjustments, all studies controlled for age and gender, while a few accounted for lifestyle attributes, thereby limiting adjustments to socio-demographic variables (e.g., highest attained level of education, income). More than half of the studies (*n* = 14) controlled for one or more air pollutants, with PM_2.5_ being the most commonly adjusted pollutant. Out of the 26 studies, the majority (*n* = 19) were classified as high-quality[6–8, 29, 30, 32–40, 42, 43, 47–49]. The remaining studies were classified as medium-quality [27, 28, 31, 41, 44–46], primarily due to limitations in outcome definition [28, 45] and/or inaccuracies in defining cases and controls [27, 44, 46].

**Table 1:** Characteristics of included studies.

Author	Year	Country	Source of transportation noise	Disease outcome	Study design and Years	Exposure Assessment and assignment	Metric	Quality assessment*
Andersson et al [7]	2020	Sweden	Road traffic	IHD, Stroke	Prospective (1970–2011)	Predictive models (Household)	Incidence, Mortality	High
Babisch et al [46]	1994	Germany	Road traffic	MI	Case–control (1994)	Physical measurement (Household)	Incidence	Medium
Babisch et al [45]	1999	United Kingdom	Road traffic	IS	Prospective (1979–1993)	Physical measurement (Household)	Incidence	Medium
Babisch et al [27]	2005	Germany	Road traffic	MI	Case–control (1998–2001)	Physical measurement (Household)	Incidence	Medium
Bai et al [48]	2020	Canada	Road traffic	MI	Retrospective (2001–2015)	Predictive models (Household)	Incidence	High
Bodin et al [28]	2016	Sweden	Road traffic	MI	Prospective (2000–2010)	Predictive models (Household)	Incidence	Medium
Carey et al [29]	2016	United Kingdom	Road traffic	MI, IS, Stroke	Prospective (2005–2011)	Predictive models (Population)	Incidence	High
Cole–Hunter et al [30]	2021	Denmark	Road traffic	IS, HS, Stroke	Prospective (1993–2014)	Predictive models (Household)	Incidence	High
Dimakopoulou et al [31]	2017	Greece	Aircraft and road traffic	MI, Stroke	Prospective (2004–2013)	Predictive models (Household)	Incidence	Medium
Grady et al [47]	2023	United States of America	Aircraft noise	CVD	Prospective (1994–2014)	Predictive models (Household)	Incidence, Mortality	High
Hao et al [32]	2022	United Kingdom	Road traffic	IHD, IS, MI, Stroke,	Prospective (2006–2021)	Predictive models (Household)	Incidence, Mortality	High
Lim et al [33]	2021	Denmark	Road traffic	MI	Prospective (1993–2014)	Predictive models (Household)	Incidence, Mortality	High
Magnoni et al [34].	2021	Italy	Road traffic	MI, IS, Stroke	Retrospective (2001–2018)	Predictive models (Household)	Incidence	High
Poulsen et al [35]	2023	Denmark	Road traffic	Stroke	Prospective (2005–2017)	Predictive models (Household)	Incidence	High
Pyko et al [8]	2019	Sweden	Aircraft, railway, and road traffic	IS, Stroke	Prospective (1990–2010)	Predictive models (Household)	Incidence	High
Roswall et al [36]	2017	Denmark	Road traffic	MI	Prospective (1997–2011)	Predictive models (Household)	Incidence, Mortality	High
Seidler et al [37]	2016	Germany	Aircraft, railway, and road traffic	IS, HS, Stroke	Case–control (2005–2010)	Physical measurement (Household)	Incidence, Mortality	High
Seidler et al [38]	2018	Germany	Aircraft, railway, and road traffic	MI	Case–control (2005–2010)	Physical measurement (Household)	Incidence	High
Selander et al [39]	2009	Sweden	Road traffic	Stroke	Case–control (1992–1994)	Predictive models (Household)	Incidence, Mortality	High
Sørensen et al [40]	2012	Denmark	Railway and road traffic	IS, HS, Stroke	Prospective (1993–2006)	Predictive models (Household)	Incidence	High
Sørensen et al [41]	2014	Denmark	Road traffic	IS	Prospective (1997–2009)	Predictive models (Household)	Incidence	Medium
Sørensen et al [42]	2021	Denmark	Road traffic	MI	Prospective (2000–2017)	Predictive models (Household)	Incidence	High
Thacher et al [43]	2022	Denmark	Aircraft, railway, and road traffic	IHD, MI	Prospective (2005–2017)	Predictive models (Household)	Incidence	High
Vienneau et al [6]	2022	Switzerland	Aircraft, railway, and road traffic	HS, IHD, IS, MI, Stroke	Prospective (2001–2015)	Predictive models (Household)	Incidence	High
Willich et al [44]	2005	Germany	Road traffic	MI	Case–control (1998–2001)	Physical measurement (Household)	Incidence	Medium
Yankoty et al [49]	2021	Canada	Road traffic	MI	Retrospective (2000–2014)	Predictive models (Household)	Incidence	High

*CVD* Cardiovascular diseases, *HS* hemorrhagic stroke, *IHD* Ischemic heart disease, *IS* Ischemic stroke, *MI* Myocardial infarction

*: “High” refers to studies scoring between 7–9 points on the Newcastle-Ottawa Scale instrument and “Medium” to studies scoring between 4-6 points on the Newcastle-Ottawa Scale instrument.

### Conventional meta-analyses and meta-regression: estimation of exposure-response relationships

**Fig. 2:**
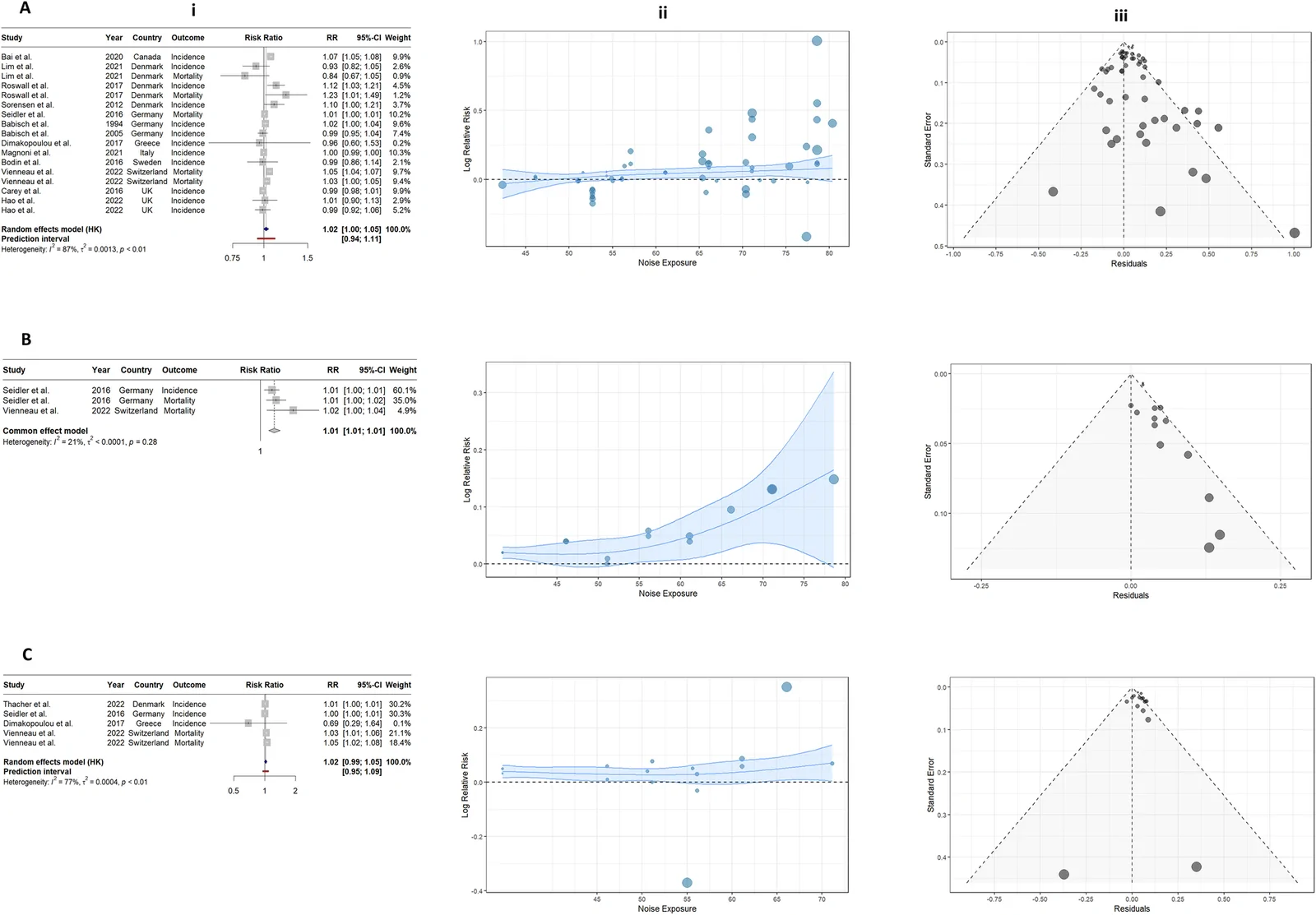
Association between long-term exposure to transportation noise sources and myocardial infarction: traditional meta-analysis framework. **A** Road traffic noise, **B** railway noise, **C** aircraft noise. (i) Forest plot of underlying relative risk (RR) data, (ii) log(RR) exposure-response function; and (iii) modified funnel plot showing the residuals (relative to 0) on the x axis and the estimated standard error and between-study heterogeneity on the y axis. Note: Meta-regression was performed using relative risk estimates associated with each noise exposure category to construct the exposure-response function (ii). Estimates from the same study were interpolated and pooled into a single estimate (per 10 dB increase), which was then pooled in the meta-analysis.

**Fig. 3:**
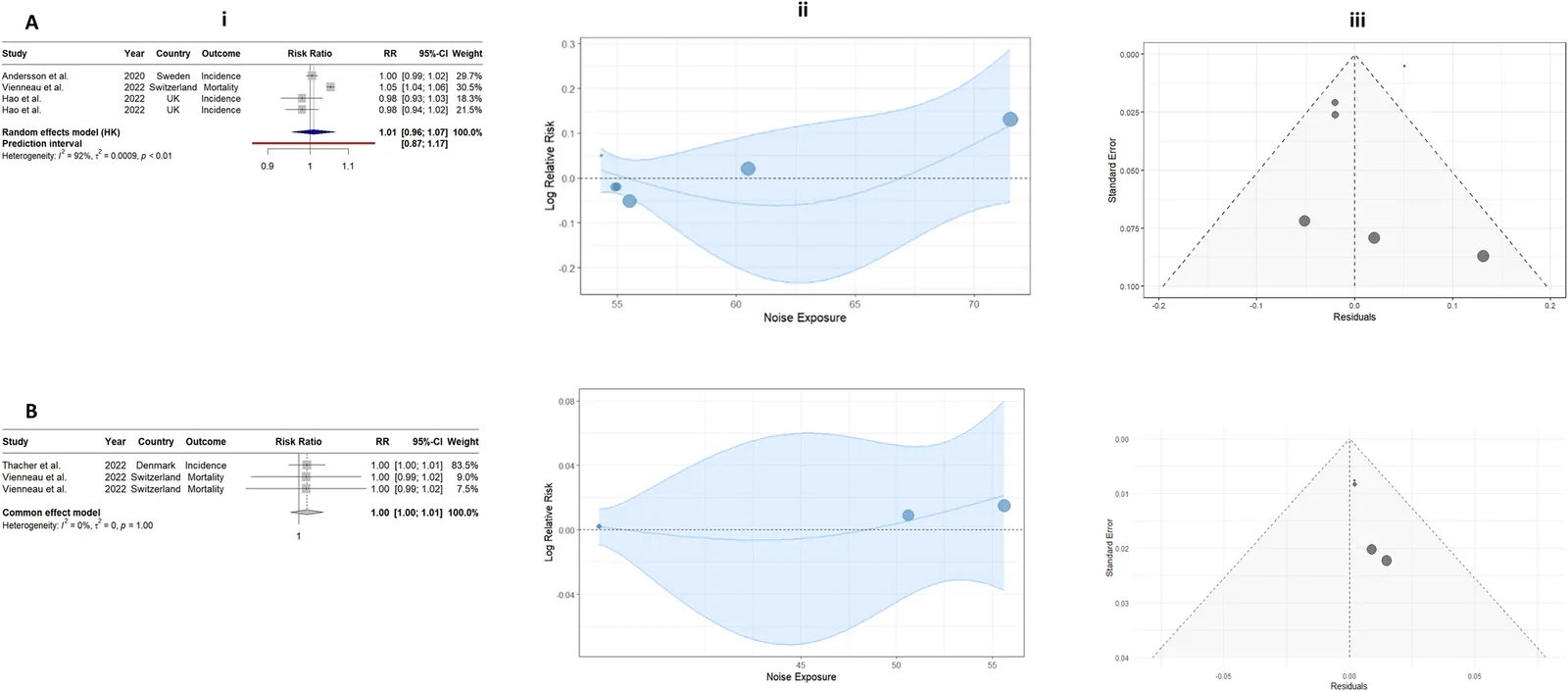
Association between long-term exposure to transportation noise sources and ischemic heart disease: traditional meta-analysis framework. **A** Road traffic noise, **B** aircraft noise. (i) Forest plot of underlying relative risk (RR) data, (ii) log(RR) exposure-response function; and (iii) modified funnel plot showing the residuals (relative to 0) on the x axis and the estimated standard error and between-study heterogeneity on the y axis. Note: Meta-regression was performed using relative risk estimates associated with each noise exposure category to construct the exposure-response function (ii). Estimates from the same study were interpolated and pooled into a single estimate (per 10 dB increase), which was then pooled in the meta-analysis.

**Fig. 4:**
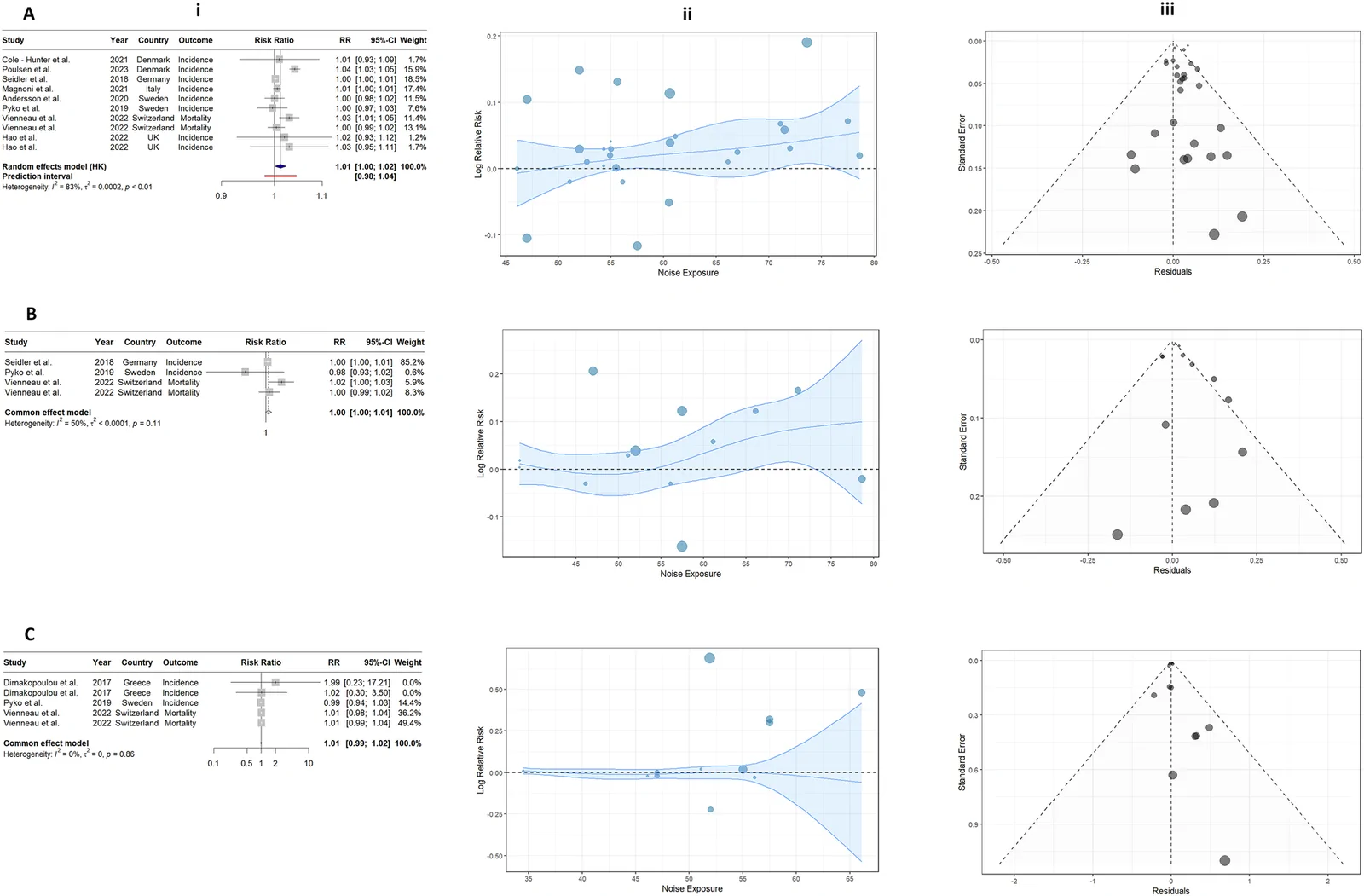
Association between long-term exposure to transportation noise sources and stroke: traditional meta-analysis framework. **A** Road traffic noise, **B** railway noise, **C** aircraft noise. (i) Forest plot of underlying relative risk (RR) data, (ii) log(RR) exposure-response function; and (iii) modified funnel plot showing the residuals (relative to 0) on the x axis and the estimated standard error and between-study heterogeneity on the y axis. Note: Meta-regression was performed using relative risk estimates associated with each noise exposure category to construct the exposure-response function (ii). Estimates from the same study were interpolated and pooled into a single estimate (per 10 dB increase), which was then pooled in the meta-analysis.

**Fig. 5:**
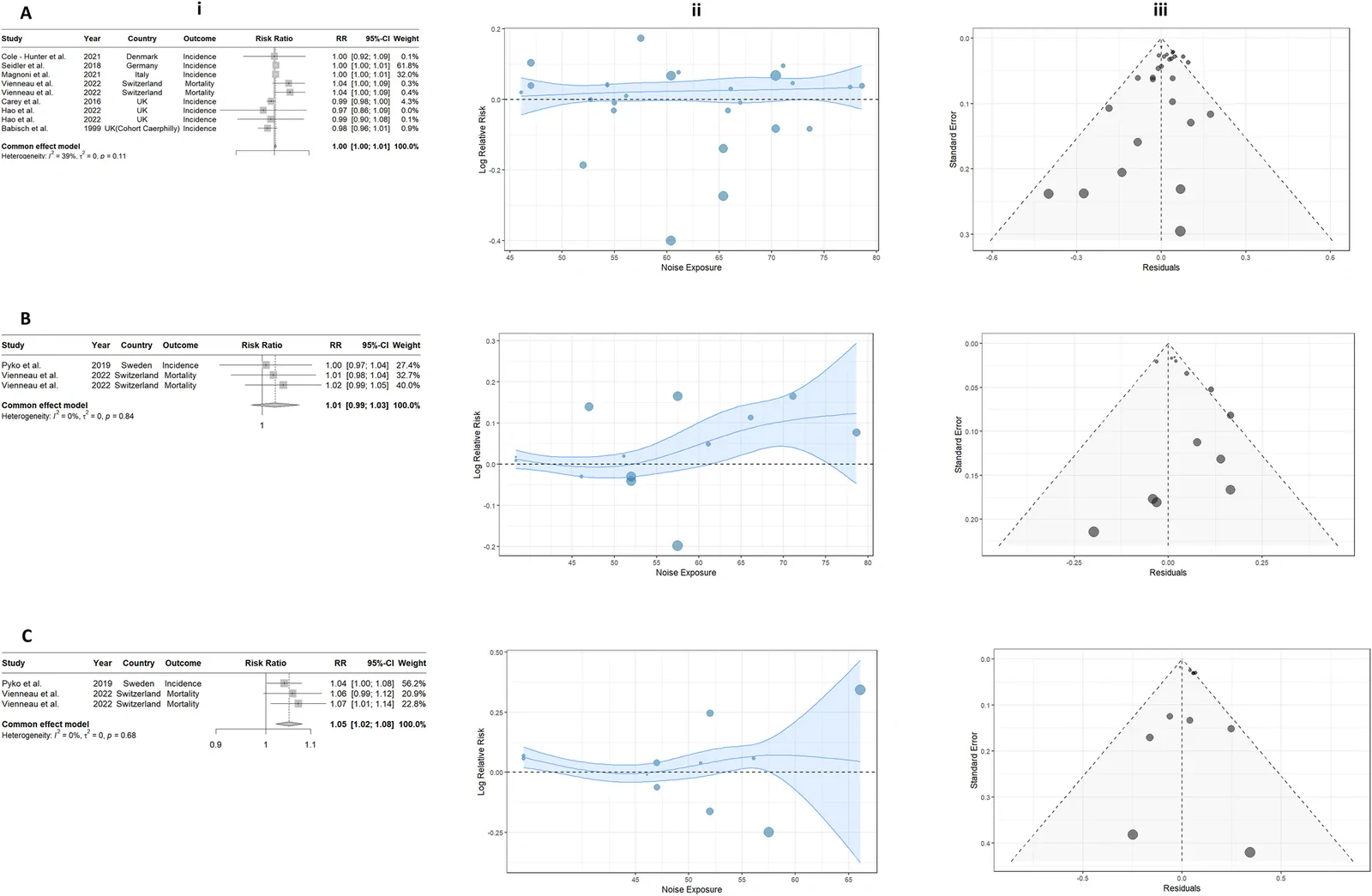
Association between long-term exposure to transportation noise sources and ischemic stroke: traditional meta-analysis framework. **A** Road traffic noise, **B** railway noise, **C** aircraft noise. (i) Forest plot of underlying relative risk (RR) data; (ii) log(RR) exposure-response function; and (iii) modified funnel plot showing the residuals (relative to 0) on the x axis and the estimated standard error and between-study heterogeneity on the y axis. Note: Meta-regression was performed using relative risk estimates associated with each noise exposure category to construct the exposure-response function (ii) Estimates from the same study were interpolated and pooled into a single estimate (per 10 dB increase), which was then pooled in the meta-analysis.

Figures 2–5 depict the results of conventional meta-analyses, including forest plots, exposure-response curves, and funnel plots for all transportation noise sources and major CVD. We found a 2, 1, and 2% increase in risk per 10 dB for myocardial infarction associated with road traffic noise (RR = 1.02, 95%CI: 1.00–1.05 95%CI, *p*-value = 0.08), railway noise (RR = 1.01, 95%CI: 1.01–1.01, *p*-value < 0.0001), and aircraft noise (RR = 1.02, 95%CI: 0.99–1.05, *p*-value = 0.14), respectively (Fig. 2A–Ci). Heterogeneity was high for the association between road traffic noise and myocardial infarction (I^2^ = 87%), with a prediction interval of 0.94–1.11, and for between aircraft noise and myocardial infarction (I^2^ = 77%), with a prediction interval of 0.95–1.09. Heterogeneity was low for the association between railway noise and myocardial infarction (I² = 21%), for which a fixed-effects model was applied (Fig. 2A–Ci). The exposure-response curves for road traffic and aircraft noise exhibited a relatively stable pattern across the exposure range, while a more pronounced increase in risk was observed above 60 dB for railway noise (Fig. 2A–Cii). There was no visual evidence of publication or reporting bias in studies determining the association between road traffic noise and myocardial infarction (Fig. 2Aiii), whereas a slight asymmetry was observed in the funnel plot for railway noise and myocardial infarction (Fig. 2Biii).

We observed a slight, not significant, increase in the risk for IHD associated with road traffic noise (RR = 1.01, 95%CI: 0.96–1.07, *p*-value = 0.64), while the association between aircraft noise and IHD was negligible (RR = 1.00, 95%CI: 1.00–1.01, *p*-value = 0.34, Fig. 3A, Bi). Heterogeneity was high for the association between road traffic noise and IHD (I² = 92%), and the prediction interval contained 1.0 (0.87–1.17). Not enough point estimates were available to determine the association between railway noise and IHD.

We also found no significant association between stroke and railway noise (RR = 1.00, 95%CI: 1.00–1.01, *p*-value = 0.08 & I^2^ = 50%) and aircraft noise (RR = 1.01, 95%CI: 0.99–1.02, p-value = 0.42 & I^2^ = 0%), while a 1% increase in risk was observed for road traffic noise (RR = 1.01, 95%CI: 1.00–1.02, *p*-value = 0.04 & I^2^ = 83%), (Fig. 4A–Ci). The greatest heterogeneity was estimated for the association between road traffic noise and stroke (I² = 83%), and the corresponding prediction interval contained 1.0 (0.98–1.04). The exposure-response curve for road traffic noise and stroke showed a gradual increase across the exposure range (Fig. 4Aii), whereas the curve for railway noise and stroke followed an exponential pattern (Fig. 4Bii). In contrast, the curve for aircraft noise and stroke remained flat, with a slight decrease beyond 55 dB (Fig. 4Cii). A 5% increase in ischemic stroke risk was associated with exposure to aircraft noise (RR = 1.05, 95%CI: 1.02–1.08, *p*-value = 0.0006 & I^2^ = 0%, Fig. 5Ci). The exposure-response curve for aircraft noise and ischemic stroke displayed a slight upward trend above 50 dB (Fig. 5Cii). We found no evidence of publication bias in studies examining the association between road traffic and stroke (Fig. 4Aiii), nor in studies on road traffic and ischemic stroke (Fig. 5Aiii).

### Burden of Proof (BoP) framework: estimation of exposure-response relationships

**Fig. 6:**
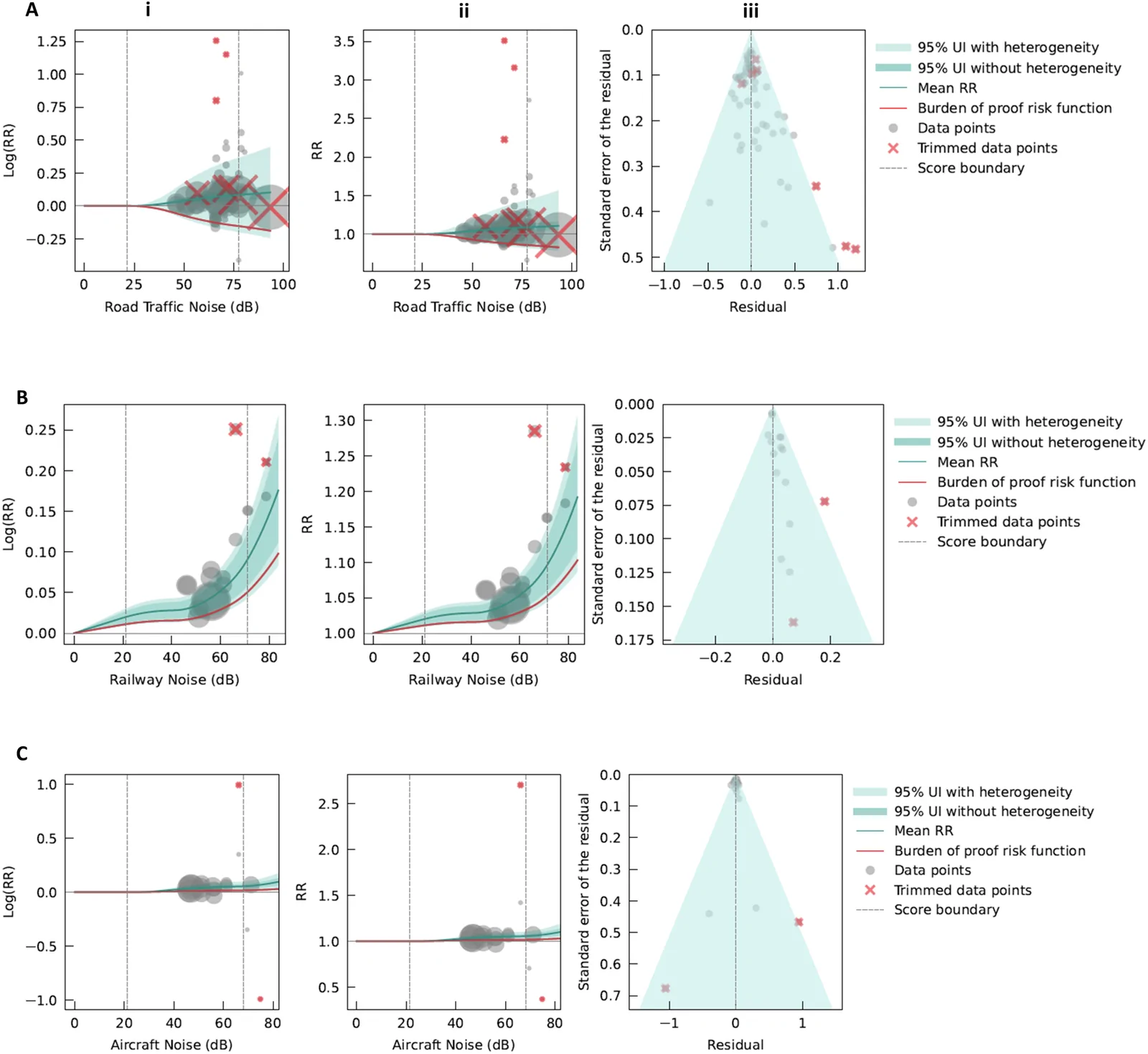
Association between long-term exposure to transportation noise sources and myocardial infarction: burden of proof framework. **A** Road traffic noise, **B** railway noise, **C** aircraft noise. (i) Log(RR) function; (ii) RR function; and (iii) modified funnel plot showing the residuals (relative to 0) on the x axis and the estimated standard error and between-study heterogeneity on the y axis.

**Fig. 7:**
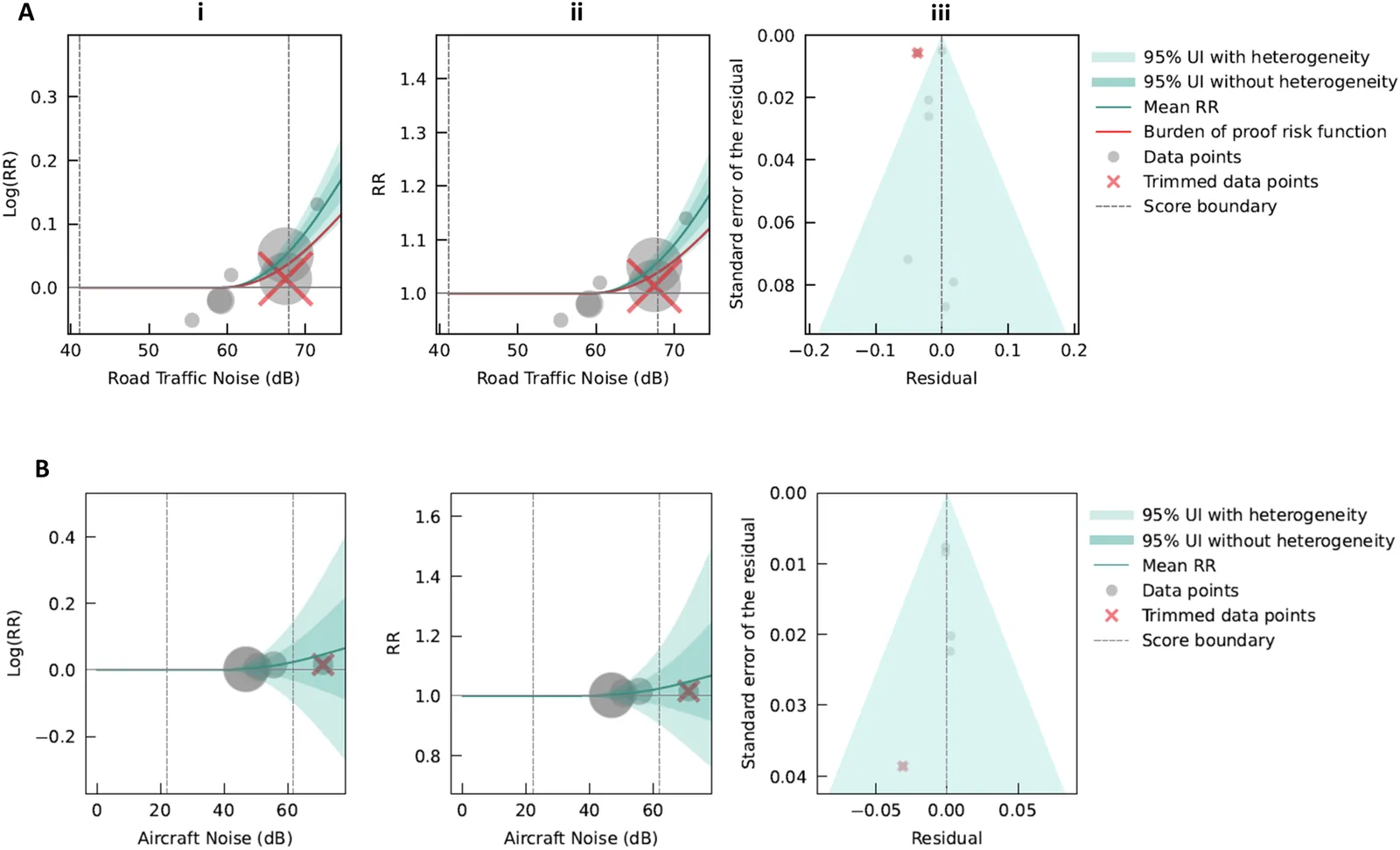
Association between long-term exposure to transportation noise sources and ischemic heart disease: burden of proof framework. **A** Road traffic noise, **B** aircraft noise. (i) log(RR) function; (ii) RR function; and (iii) modified funnel plot showing the residuals (relative to 0) on the x axis and the estimated standard error and between-study heterogeneity on the y axis.

**Fig. 8:**
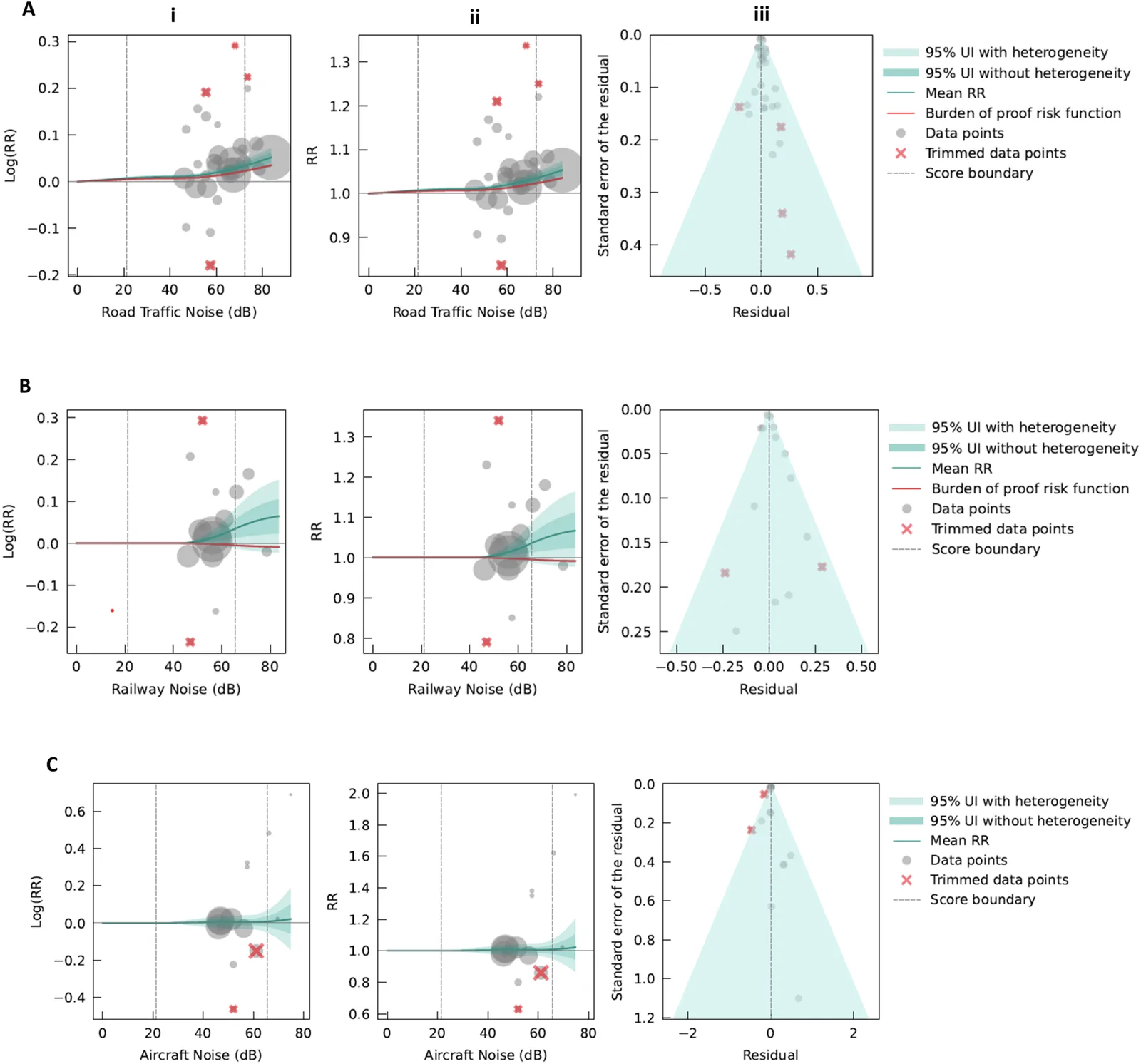
Association between long-term exposure to transportation noise sources and stroke: burden of proof framework. **A** Road traffic noise, **B** railway noise, **C** aircraft noise. (i) Log(RR) function; (ii) RR function; and (iii) modified funnel plot showing the residuals (relative to 0) on the x axis and the estimated standard error and between-study heterogeneity on the y axis.

**Fig. 9:**
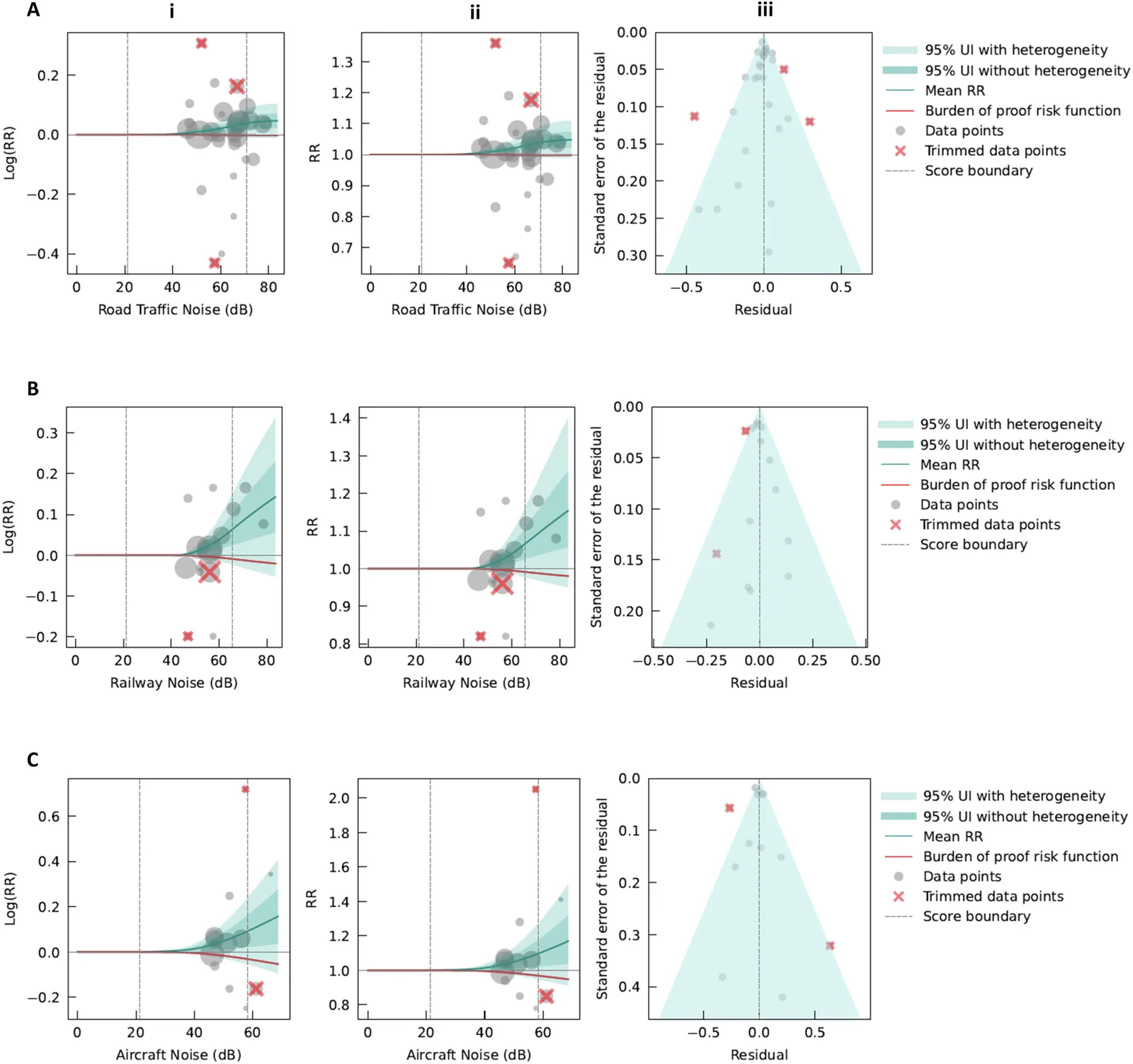
Association between long-term exposure to transportation noise sources and ischemic stroke: burden of proof framework. **A** Road traffic noise, **B** railway noise, **C** aircraft noise. (i) Log(RR) function; (ii) RR function; and (iii) modified funnel plot showing the residuals (relative to 0) on the x axis and the estimated standard error and between-study heterogeneity on the y axis.

Table 2 shows the estimates of all identified risk-outcome pairs. Figures 6–9 illustrate the results of the BoP framework, including log(RR) functions, RR functions, and funnel plots, for transportation noise sources and major CVD outcomes. Among the assessed risk-outcome pairs, four had a two-star rating, suggesting that exposure to transportation noise sources increases the risk of a given CVD outcome by 0-15% which can be interpreted as weak evidence. For example, myocardial infarction showed a two-star association with railway noise (RR = 1.16, 95%UI inclusive between-study heterogeneity: 1.07–1.26, ROS = 0.02, BPRF = 1.04) and with aircraft noise (RR = 1.09, 95%UI inclusive between-study heterogeneity: 1.01–1.18, ROS = 0.01, BPRF = 1.02), Table 2. Among the two-star associations, the largest number of studies was found for the association between road traffic noise and stroke (*n* = 10 studies, RR = 1.05, 95%UI inclusive between-study heterogeneity: 1.03–1.07, ROS = 0.01, BPRF = 1.02, Table 2 & Fig. 8A).

**Table 2:** Strength of the evidence for the relationship between transportation noise sources and major cardiovascular disease outcomes.

CVD outcome	Transportation noise source	RR per 10 dB (95%UI without between-study heterogeneity)	RR per 10 dB (95%UI with between-study heterogeneity)	BPRF	ROS	Star rating	Publication bias	Number of studies	Risk-outcome pair in GBD 2023 study
Myocardial Infarction	Road traffic noise	1.10 (1.05–1.15)	1.10 (0.79–1.56)	0.87	–0.07	✩	No	15	No
	Railway noise	1.16 (1.10–1.22)	1.16 (1.07–1.26)	1.04	0.02	✩✩	Yes	2	No
	Aircraft noise	1.09 (1.06–1.12)	1.09 (1.01–1.18)	1.02	0.01	✩✩	No	4	No
Ischemic Heart Disease	Road traffic noise	1.27 (1.22–1.33)	1.27 (1.16–1.41)	1.00	0.00	✩✩	No	3	No
	Railway noise	–	–	–	–	–	–	–	No
	Aircraft noise	1.07 (0.91–1.25)	1.07 (0.74–1.55)	N/A	N/A		No	2	No
Stroke	Road traffic noise	1.05 (1.04–1.06)	1.05 (1.03–1.07)	1.02	0.01	✩✩	No	10	No
	Railway noise	1.06 (1.02–1.10)	1.06 (0.98–1.16)	1.00	0.00	✩	No	3	No
	Aircraft noise	1.01 (0.96–1.07)	1.01 (0.90–1.15)	N/A	N/A		No	4	No
Ischemic Stroke	Road traffic noise	1.05 (1.02–1.07)	1.05 (0.99–1.11)	1.00	0.00	✩	No	10	No
	Railway noise	1.14 (1.05–1.22)	1.14 (0.95–1.37)	1.00	0.00	✩	No	3	No
	Aircraft noise	1.15 (1.11–1.26)	1.15 (0.92–1.45)	0.98	-0.01	✩	No	3	No

*BPRF* burden of proof risk function, *CVD* cardiovascular disease, *N/A* not applicable, *ROS* risk-outcome score, *RR* relative risk, *UI* uncertainty interval.

The reported RR and its 95% UI reflect the risk of developing the outcome of interest (i.e., CVD outcome) among individuals who have been exposed to road traffic, railway, or aircraft noise, relative to that of those who have not been exposed to these environmental stressors. We report the 95%UI without and with between-study heterogeneity. The BPRF is calculated for each risk-outcome pair that showed a significant association at the 0.05 level when between-study heterogeneity is not considered. The ROS is calculated as the signed value of log(BPRF) divided by two. Negative ROS indicate that the evidence of the association is very weak and inconsistent. The ROS and BPRF are transformed into star-rating categories ranging from one to five; a one-star (ROS value ≤ 0.0) indicates weak evidence of association, while a five-star (ROS value > 0.62) indicates very strong evidence of association.

The BPRF analysis yielded a one-star rating for five risk-outcome pairs (e.g., aircraft noise and ischemic stroke, RR = 1.15, 95%UI inclusive between-study heterogeneity: 0.92–1.45, ROS = −0.01, BPRF = 0.98, Table 2), indicating that exposure to transportation noise sources is weakly associated with certain CVD outcomes. Among the one-star associations, the largest number of studies was found for the association between road traffic noise and myocardial infarction (*n* = 15 studies, RR = 1.10, 95%UI inclusive between-study heterogeneity: 0.79–1.56, ROS = −0.07, BPRF = 0.87, Table 2) and for road traffic noise and ischemic stroke (*n* = 10 studies, RR = 1.05, 95%UI inclusive between-study heterogeneity: 0.99–1.11, ROS = 0.00, BPRF = 1.00, Table 2). The RR function for railway noise and IHD could not be estimated due to insufficient data, while ROS and BPRF estimation for aircraft noise and IHD and stroke was not feasible due to non-detectable between-study heterogeneity (I^2^ = 0%). None of the covariates examined was significant across the assessed risk-outcome pairs.

The mean risk curves either increased sharply (e.g., railway noise and myocardial infarction, Fig. 6B; road traffic noise and IHD, Fig. 7A; railway noise and ischemic stroke, Fig. 9B), increased steadily (e.g., road traffic noise and myocardial infarction, Fig. 6Aii & road traffic noise and ischemic stroke, Fig. 9Aii) or plateaued (e.g., aircraft noise and myocardial infarction, Fig. 6Cii & aircraft noise and stroke, Fig. 8Cii). Most funnel plots (Figs. 6–9iii) showed that, after trimming, the residual standard error—reflecting both variance and between-study heterogeneity—fell within the expected range of the model. However, slight asymmetry was observed in the funnel plot for railway noise and myocardial infarction (Fig. 6Biii).

## Discussion

This is the first study to apply the conventional meta-regression and MR-BRT approach to quantify and evaluate associations between long-term exposure to transportation noise sources (i.e., road traffic, railway, and aircraft) and combined risk of non-fatal and fatal CVD outcomes, including myocardial infarction, IHD, stroke, and ischemic stroke. Of the 26 studies included, most were cohort studies conducted in Europe and focused on road traffic noise. Long-term exposure to road traffic and railway noise was significantly associated with increased risk of stroke and myocardial infarction, respectively. These two risk-outcome pairs showed the strongest and most consistent associations across both the conventional meta-regression and MR-BRT approach; however, the strength of evidence was classified as weak. Differences in curve shapes were observed, with the conventional meta-regression approach allowing the curves to fluctuate in response to the input estimates, and the MR-BRT approach producing smoother and more plausible exposure-response curves.

This study shows that substantial new evidence on transportation noise sources and major CVD outcomes has emerged since the publication of the 2018 WHO Environmental Noise Guidelines [5]. Our conventional meta-analysis yielded a pooled RR estimate of 1.01 (95%CI: 1.00–1.02) per 10 dB L_den_ for the association between road traffic noise and stroke. This risk is lower compared to a recent systematic review and meta-analysis that reported a RR estimate of 1.025 (95%CI: 1.009–1.041) for road traffic noise and stroke incidence [50]. The difference in RR likely reflects variations in study inclusion criteria and/or differences in weighting approaches. However, when applying the MR-BRT approach, which accounts for study-specific deviations and down-weights inconsistent evidence, we found more robust evidence of the association of road traffic noise with stroke (RR = 1.05, 95%UI: 1.03–1.07).

Furthermore, our conventional meta-analysis identified a positive association between road traffic noise and myocardial infarction (RR = 1.02, 95%CI: 1.00–1.05), whereas the MR-BRT approach produced a higher estimate (RR = 1.10, 95%UI: 0.79–1.56) for this association. The wider 95%UI, compared with those (typically) reported in traditional meta-analyses, reflects the inclusion of unexplained heterogeneity beyond between-study heterogeneity in mean effects [13, 16]. Our conventional meta-analysis results are consistent in direction and magnitude with those of previous meta-analyses reporting modest but statistically significant associations; however, differences in study inclusion criteria and model adjustments may limit direct comparability [50, 51]. For example, inconsistent adjustments for correlated traffic-related exposures have previously been found. The major challenge is that transportation noise and air pollution correlate, reflecting that traffic is the main source of both exposures. Eminson et al. found no strong evidence that air pollution confounds the associations between traffic noise and CVD outcomes, whereas Pershagen et al. found that PM_2.5_, but not NO_2_, confounded the traffic noise-related associations [50, 52]. One possible explanation for these inconsistent findings is that disentangling the effects of both transportation noise and air pollution exposures requires a high-quality assessment of both in order to avoid one exposure being estimated more precisely than the other. Some studies have therefore recommended deriving two-pollutant effect estimates when correlations between air pollutants are low [53, 54]. Inclusion of highly correlated air pollutants when deriving two-pollutant effect estimates can attenuate the *true* long-term effects of noise. Sørensen et al. reported that long-term exposure to NO_2_ and road traffic noise was associated with increased risk of CVD outcomes in both single- and two-pollutant models, while Osborne et al. reported that combined exposure to both noise and air pollution was associated with increased risk of major adverse CVD events [55, 56]. Experimental studies are needed to further examine the effects of simultaneous exposure to traffic noise and air pollution and to address how these exposures interact at a mechanistic level.

Consistent with previous systematic reviews and meta-analyses, we identified only two studies examining the association between railway noise and myocardial infarction [57–59]. Our conventional meta-analysis showed a minimal effect of a 10 dB L_den_ increase in railway noise exposure on the combined risk of myocardial infarction incidence and mortality (RR = 1.01, 95%CI: 1.01–1.01), whereas the MR-BRT approach yielded a RR estimate of 1.16 (95%UI: 1.07–1.26). The estimate derived from the conventional meta-analysis was lower than the hazard ratio (HR) reported for fatal myocardial infarction in a large Danish and Swedish cohort study (HR = 1.08, 95%CI: 0.99–1.17), whereas the one derived from the MR-BRT was higher [60].

We also found aircraft noise as a potential environmental risk factor for the combined risk of ischemic stroke incidence and mortality; our conventional meta-analysis yielded a RR estimate of 1.05 (95%CI: 1.02–1.08), whereas the MR-BRT yielded a RR estimate of 1.15 (95%UI: 1.11–1.26). Ischemic stroke accounts for approximately two-thirds of all incident strokes and has previously been reported to be significantly associated with transportation noise sources, such as road traffic noise [61, 62]. Noise-induced endothelial dysfunction is a recognized biological mechanism that increases thrombotic risk and contributes to the development of ischemic stroke [63]. In a nationwide cohort study from Switzerland, Héritier et al. found significant positive associations of both road traffic and aircraft noise with ischemic stroke mortality, but not with stroke in general [10]. Similar findings have also been reported in the same cohort with longer follow-up time [6].

This study revealed marked differences between the conventional meta-regression and the MR-BRT approach. These differences were particularly evident in risk-outcome pairs with limited underlying evidence, which demonstrates the strength of the MR-BRT approach in generating more robust and plausible effect estimates when input data are sparse or scattered. Differences were further evident in the exposure-response curves: those derived using natural cubic splines were non-smooth and less interpretable compared to those derived with the MR-BRT approach. Using Bayesian priors, the MR-BRT approach reduces the influence of outliers, producing smoother curves and showing a consistent increase in risk, particularly at higher exposure levels (e.g., road traffic noise-IHD and aircraft noise-IHD pairs, Fig. 3A, Bii & Fig. 7A, Bii). Future conventional meta-regression analyses should acknowledge the methodological limitations of natural cubic splines in modeling exposure-response functions, particularly when comparing region-specific curves that may be affected by differences in noise exposure levels, study design, and/or confounding factors.

In this study, we pooled estimates from studies reporting either disease incidence or mortality. This approach is consistent with the BoP framework, which supports a unified risk estimate under specific conditions: when a common biological mechanism is presumed, when only incidence or mortality data are available, or when no statistically significant difference is observed between the two outcomes [16, 64]. Chronic stress, sleep disturbance, vascular inflammation, and endothelial dysfunction have been proposed as key biological pathways linking environmental noise to CVD [58, 65]. Although these mechanisms likely contribute to both non-fatal and fatal events, mortality is also influenced by additional factors, such as comorbidities and access to healthcare. Thus, combining morbidity and mortality outcomes in meta-analyses may mask differential risks. Nevertheless, in our stratified analyses, we observed similar pooled RR for road traffic noise and non-fatal myocardial infarction (RR = 1.04, 95%CI: 0.98-1.11, Supplementary Fig. S3, Supplementary material pp 18) and fatal myocardial infarction (RR = 1.04, 95%CI: 1.03-1.05, Supplementary Fig. S4, Supplementary material pp 18). We also observed that road traffic noise was the most frequently studied transportation noise source, and that its associations with CVD outcomes exhibited greater between-study heterogeneity than those observed for railway and aircraft noise. This heterogeneity may reflect the broader range of geographical and urban settings in which road traffic noise has been studied, leading to variation in study population composition, the quality of the noise exposure assessment, and the extent and quality of adjustment as well as other unmeasured sources of bias. By contrast, data on aircraft and railway noise remain limited primarily due to monitoring challenges [66, 67]. These challenges increase the risk of underrepresenting affected populations. For instance, railway noise is a relatively rare exposure, affecting a limited proportion of the population. We also observed substantial variability across studies in the noise indicators used to assess participant exposure, including methods used to quantify exposure and the predictive models used to map noise exposure across urban grids [68]. L_den_ should continue to be regarded as the preferred noise indicator for measuring exposure, as its standardized nature allows for more consistent comparisons of findings [18]. In addition, most of the included studies were conducted in Denmark, Germany, and Sweden—countries that have long recognized environmental noise as a significant public health concern. More efforts, including regulatory and legislative changes, are needed across the European region in order to achieve the European Union’s zero pollution action target: *“reducing the share of people chronically disturbed by transport noise by 30%”* [69, 70].

This study must be considered within its limitations. First, the small number of studies per risk–outcome pair precluded the derivation of stratified exposure-response functions by age, gender, and other covariates. Second, due to the relatively small number of studies analyzed per risk-outcome pair, it was not feasible to disentangle whether, or to what extent, confounders drove differences between studies. Third, this study may have introduced a potential bias by limiting inclusion to studies published in English. Fourth, this study included a limited number of studies that used physical measurements to assess exposures, which precluded sensitivity analyses comparing the impact of different assessment methods (e.g. physical measurement *versus* predictive models) on the association between transportation noise sources and cardiovascular disease outcomes. Nevertheless, future systematic reviews and meta-analyses should address this, as differences in exposure assessment methods may influence overall effect estimates. Although the two approaches used in the present study relied on trimmed effect estimates, differences in study weighting (i.e., inverse-variance weighting in conventional meta-analysis *versus* penalized regression in MR-BRT) and in the handling of residual heterogeneity likely contributed to the discrepancy in effect estimates. Our conventional meta-analysis incorporated trimmed data primarily to enable direct comparison with BPRF estimates, and also because conventional meta-analyses often overestimate risks by pooling heterogeneous data without adequately addressing outliers and study variability. The BoP framework addresses these issues, producing more precise, reliable, and conservative risk estimates that are also more robust. Given these advantages, we recommend using BoP framework risk functions in future transportation noise-specific burden-of-disease assessments. Our study contributes to the existing literature on transportation noise-related risk functions, which are quintessential to determining the burden of disease attributable to transportation noise. A landmark study that provides such population health data is the GBD study. While the most recent iteration of the GBD study has expanded the range of environmental risk factors included, such as the inclusion of ambient NO_2_ and non-optimal temperatures, transportation noise is currently not covered, even though it may represent a significant contributor to the global disease burden [16, 71]. Our findings using the BoP framework on transportation noise and major CVD outcomes advance the integration of an additional environmental risk factor and propose new risk-outcome pairs for potential inclusion in the GBD study.

## Supplementary information

## Data Availability

The appendix provides the full list of articles that informed this systematic review and meta-analysis, information about the articles, and overview of the study methodology and performed statistical analyses. All code used for the conventional meta-analyses (github.com/egonzato/noisecvds_meta-analysis) and for the MR-BRT (github.com/ihmeuw-msca/burden-of-proof) are publicly available.
