# Supplementary material for: Exposure-response relationship between transportation noise and cardiovascular disease outcomes: a systematic review and meta-regression analysis
Source: J Expo Sci Environ Epidemiol. 2026 Apr 6;36(5):998–1012. doi: 10.1038/s41370-026-00856-9 (PMC13538067; doi:10.1038/s41370-026-00856-9)
Supplement: Supplementary file 1 [file 41370_2026_856_MOESM1_ESM.docx]

**Supplementary Material**

Supplement Material: Exposure-response relationship between transportation noise and cardiovascular disease outcomes: a systematic review and meta-regression analysis

**Preamble**

This appendix provides further methodological details and supplementary descriptive results for *“Exposure-response relationship between transportation noise and cardiovascular disease outcomes: a systematic review and meta-regression analysis”.* This study complies with both the Preferred Reporting Items for Systematic Reviews and Meta-Analyses (PRISMA) and the Guidelines for Accurate and Transparent Health Estimates Reporting (GATHER) recommendations.^1,2^

**Table of Contents**

**Preamble……………………………………………………………………………………………………………… 2**

**Section 1: Systematic review: exposure and outcome definitions……………………………..………..…………..4**

1.1 Search strategy….……………………………………………………….………………………………..4

1.2 Exposure and Outcome definitions….………………………………………………………….…………5

1.2.1 Case definition and ICD codes for the disease outcome examined.......………….…………….5

1.2.2 Description of included environmental noise indicators………………………...…………….6

1.3 Definitions of the data extraction items……………………………………………………..…………….6

1.4 Standard bias covariates.……………………………………………………………….………………..13

1.5 Exposure Assessment and Assignment..…....……………………………………………………………13

**Section 2: Meta-Analysis: supplementary methods…..………………………………………………………..…..14**

2.1 Data pre-processing..…..…………………………………………………………………………..…….14

2.2 Conventional Meta-Analysis: methods……………………….………….…………………………..….14

**Section 3: Systematic review: supplementary results…..…..………………………..…………………….…..…..15**

3.1 Characteristics of included studies……..………………………………………………………..………15

3.1.1 Number of included studies by geographical location………………..........…………………17

3.1.2 Number of identified risk-outcome pairs………………………………..……………………17

**Section 4: Meta-Analysis: supplementary results……………..……………………………….…………………..18**

4.1 Supplementary results on conventional meta-analysis………………………………………………….18

**Section 5: Research and reporting practices………………………………………………………………………21**

5.1 Statement of PRISMA 2020 compliance..…….…….…………………………………………………...21

5.2 Statement of GATHER compliance...……….………………………………………………..………….23

**Section 6: References….…………………………………………………………………………..…………..……..24**

**Section 1: Systematic review: exposure and outcome definitions**

1.1 Search strategy

Embase

('traffic noise'/de OR 'aircraft noise'/de OR ((noise/exp OR 'noise pollution'/exp OR 'noise exposure'/de) AND ('traffic and transport'/exp)) OR (((traffic* OR transport* OR aircraft* OR plane OR planes OR airplane* OR aeroplane* OR airline OR airlines OR rail* OR train OR trains OR road* OR car OR cars OR aviation* OR urban OR airport* OR vehicl* OR jet OR jets) NEAR/6 (noise*))):ab,ti,kw) AND ('ischemic heart disease'/exp OR 'cerebrovascular accident'/de OR 'coronary artery disease'/exp OR 'heart disease'/de OR 'cardiovascular disease'/de OR (((ischemic* OR ischaemic* OR coronar* OR atherosclero*) NEAR/6 (heart* OR cardiac* OR cardial) NEAR/6 (diseas*)) OR ((acute) NEAR/6 (coronar*) NEAR/6 (syndrom*)) OR stroke* OR ((cerebrovascular) NEAR/3 (accident*)) OR ((angina) NEAR/3 (pector*)) OR ((myocardia* OR heart* OR cardia*) NEAR/3 (infarct* OR attack*)) OR IHD OR ((cardiovascular OR cerebrovascular*) NEAR/3 (diseas*)) OR ((myocardia* OR cardia*) NEAR/3 (ischem*)) OR ((cardiogen*) NEAR/3 (shock*)) OR ((atherosclerot*) NEAR/6 (heart* OR cardiac OR cardial) NEAR/6 (diseas*)) OR ((coronar*) NEAR/6 (arter*) NEAR/6 (diseas*))):ab,ti,kw) NOT (([Conference Abstract]/lim OR [Conference Review]/lim) AND [1800-2020]/py) NOT ([animals]/lim NOT [humans]/lim)

Medline

(Noise, Transportation/ OR ((exp Noise/) AND (exp Transportation/ OR Traffic-Related Pollution/)) OR (((traffic* OR transport* OR aircraft* OR plane OR planes OR airplane* OR aeroplane* OR airline OR airlines OR rail* OR train OR trains OR road* OR car OR cars OR aviation* OR urban OR airport* OR vehicl* OR jet OR jets) ADJ6 (noise*))).ab,ti,kf.) AND (exp Myocardial Ischemia/ OR Stroke/ OR Coronary Artery Disease/ OR Heart Diseases/ OR Cardiovascular Diseases/ OR (((ischemic* OR ischaemic* OR coronar* OR atherosclero*) ADJ6 (heart* OR cardiac* OR cardial) ADJ6 (diseas*)) OR ((acute) ADJ6 (coronar*) ADJ6 (syndrom*)) OR stroke* OR ((cerebrovascular) ADJ3 (accident*)) OR ((angina) ADJ3 (pector*)) OR ((myocardia* OR heart* OR cardia*) ADJ3 (infarct* OR attack*)) OR IHD OR ((cardiovascular OR cerebrovascular*) ADJ3 (diseas*)) OR ((myocardia* OR cardia*) ADJ3 (ischem*)) OR ((cardiogen*) ADJ3 (shock*)) OR ((atherosclerot*) ADJ6 (heart* OR cardiac OR cardial) ADJ6 (diseas*)) OR ((coronar*) ADJ6 (arter*) ADJ6 (diseas*))).ab,ti,kf.) NOT ((news OR congres* OR abstract* OR book* OR chapter* OR dissertation abstract*).pt. AND 1800:2020.(sa_year).) NOT (exp Animals/ NOT Humans/)

Cochrane

((((traffic* OR transport* OR aircraft* OR plane OR planes OR airplane* OR aeroplane* OR airline OR airlines OR rail* OR train OR trains OR road* OR car OR cars OR aviation* OR urban OR airport* OR vehicl* OR jet OR jets) NEAR/6 (noise*))):ab,ti) AND ((((ischemic* OR ischaemic* OR coronar* OR atherosclero*) NEAR/6 (heart* OR cardiac* OR cardial) NEAR/6 (diseas*)) OR ((acute) NEAR/6 (coronar*) NEAR/6 (syndrom*)) OR stroke* OR ((cerebrovascular) NEAR/3 (accident*)) OR ((angina) NEAR/3 (pector*)) OR ((myocardia* OR heart* OR cardia*) NEAR/3 (infarct* OR attack*)) OR IHD OR ((cardiovascular OR cerebrovascular*) NEAR/3 (diseas*)) OR ((myocardia* OR cardia*) NEAR/3 (ischem*)) OR ((cardiogen*) NEAR/3 (shock*)) OR ((atherosclerot*) NEAR/6 (heart* OR cardiac OR cardial) NEAR/6 (diseas*)) OR ((coronar*) NEAR/6 (arter*) NEAR/6 (diseas*))):ab,ti) NOT "conference abstract":kw

Web of Science

TS=(((((traffic* OR transport* OR aircraft* OR plane OR planes OR airplane* OR aeroplane* OR airline OR airlines OR rail* OR train OR trains OR road* OR car OR cars OR aviation* OR urban OR airport* OR vehicl* OR jet OR jets) NEAR/5 (noise*)))) AND ((((ischemic* OR ischaemic* OR coronar* OR atherosclero*) NEAR/5 (heart* OR cardiac* OR cardial) NEAR/5 (diseas*)) OR ((acute) NEAR/5 (coronar*) NEAR/5 (syndrom*)) OR stroke* OR ((cerebrovascular) NEAR/2 (accident*)) OR ((angina) NEAR/2 (pector*)) OR ((myocardia* OR heart* OR cardia*) NEAR/2 (infarct* OR attack*)) OR IHD OR ((cardiovascular OR cerebrovascular*) NEAR/2 (diseas*)) OR ((myocardia* OR cardia*) NEAR/2 (ischem*)) OR ((cardiogen*) NEAR/2 (shock*)) OR ((atherosclerot*) NEAR/5 (heart* OR cardiac OR cardial) NEAR/5 (diseas*)) OR ((coronar*) NEAR/5 (arter*) NEAR/5 (diseas*)))) NOT ((animal* OR rat OR rats OR mouse OR mice OR murine OR dog OR dogs OR canine OR cat OR cats OR feline OR rabbit OR cow OR cows OR bovine OR rodent* OR sheep OR ovine OR pig OR swine OR porcine OR veterinar* OR chick* OR zebrafish* OR baboon* OR nonhuman* OR primate* OR cattle* OR goose OR geese OR duck OR macaque* OR avian* OR bird* OR fish*) NOT (human* OR patient* OR women OR woman OR men OR man))) NOT (DT=(Meeting Abstract OR Meeting Summary) AND py=(1800-2020))

Scopus

TITLE-ABS-KEY(((((traffic* OR transport* OR aircraft* OR plane OR planes OR airplane* OR aeroplane* OR airline OR airlines OR rail* OR train OR trains OR road* OR car OR cars OR aviation* OR urban OR airport* OR vehicl* OR jet OR jets) W/5 (noise*)))) AND ((((ischemic* OR ischaemic* OR coronar* OR atherosclero*) W/5 (heart* OR cardiac* OR cardial) W/5 (diseas*)) OR ((acute) W/5 (coronar*) W/5 (syndrom*)) OR stroke* OR ((cerebrovascular) W/2 (accident*)) OR ((angina) W/2 (pector*)) OR ((myocardia* OR heart* OR cardia*) W/2 (infarct* OR attack*)) OR IHD OR ((cardiovascular OR cerebrovascular*) W/2 (diseas*)) OR ((myocardia* OR cardia*) W/2 (ischem*)) OR ((cardiogen*) W/2 (shock*)) OR ((atherosclerot*) W/5 (heart* OR cardiac OR cardial) W/5 (diseas*)) OR ((coronar*) W/5 (arter*) W/5 (diseas*)))) AND NOT ((animal* OR rat OR rats OR mouse OR mice OR murine OR dog OR dogs OR canine OR cat OR cats OR feline OR rabbit OR cow OR cows OR bovine OR rodent* OR sheep OR ovine OR pig OR swine OR porcine OR veterinar* OR chick* OR zebrafish* OR baboon* OR nonhuman* OR primate* OR cattle* OR goose OR geese OR duck OR macaque* OR avian* OR bird* OR fish*) AND NOT (human* OR patient* OR women OR woman OR men OR man))) AND NOT DOCTYPE(ab)

Global Index Medicus

(noise*) AND (heart* OR cardiac OR cardial OR angina* OR myocardial* OR cardiovacular*)

Google Scholar

'traffic|transportation|aircraft|railway|railroad|aviation noise' 'ischemic|coronary heart|cardiac disease'|'acute coronary syndrome'|stroke|'cerebrovascular accident'|'angina pectoris'|'heart|cardiac infarct|attack'|'cardiovascular|cerebrovascular disease'

1.2 **Exposure and Outcome definitions**

**1.2.1 Table S1: Case definition and ICD codes for the disease outcomes examined**

Following the International Classification of Diseases (ICD) diagnostic criteria, we defined non-fatal (measured by incidence) and fatal (measured by mortality) cases for myocardial infraction, ischemic heart disease (IHD), stroke, and ischemic stroke cases. The case definitions for the disease outcomes examined are detailed in Table S1.^3^

| **Disease outcome** | **Case definition** | **ICD code** |
| --- | --- | --- |
| Myocardial Infarction | Definite and possible myocardial infraction according to the fourth universal definition of myocardial infarction:  a. When there is clinical evidence of myocardial necrosis in a clinical setting consistent with myocardial ischemia; or  b. Detection of a rise and/or fall of cardiac biomarker values and with at least one of the following: i) symptoms of ischemia, ii) new or presumed new ST-segment-T wave changes or new left bundle branch block, iii) development of pathological Q waves in the ECG, iv) imaging evidence of new loss of viable myocardium or new regional wall motion abnormality, or v) identification of an intracoronary thrombus by angiography or autopsy; or  c. Sudden (abrupt) unexplained cardiac death, involving cardiac arrest or no evidence of a non-coronary cause of death. | ICD9: 410  ICD10: I21-I22 |
| Ischemic Heart Disease | Disease that limits the supply of blood to the heart. IHD is typically due to the narrowing of the coronary arteries, usually due to atherosclerosis, which limits blood flow. The Global Burden of Disease (GBD) study estimates IHD as the aggregate of discrete sequelae, consisting of myocardial infarction (heart attacks), angina (stable ischemic heart disease manifesting as chest pain), or ischemic cardiomyopathy (heart failure due to IHD). | ICD-9: 411-414.9 (excluding angina pectoris; ICD-9: 413)  ICD-10: I24-I25.9 (excluding angina pectoris; ICD-10: I20) |
| Stroke | Following the WHO criteria, stroke is defined as rapidly developing clinical signs of focal (or less commonly global) disturbance of cerebral function lasting more than 24 hours or leading to death with no apparent cause other than that of vascular origin. | ICD9: 430-438.9, 436  ICD10: I60-I69.8 |
| Ischemic Stroke | Ischemic strokes are characterized by occlusion of blood flow to part of the brain due to hypoperfusion, most commonly due to a thrombus or embolism. It is defined as an episode of neurological dysfunction caused by focal cerebral, spinal, or retinal infarction. | ICD-9: 433-435.9, 437.0-437.1, 437.5- 437.8  ICD-10: G45-G46.8, I63-I63.9, I65-I66.9, I67.2-I67.3, I67.5-I67.6, I69.3 |

**1.2.2 Table S2: Description of included environmental noise indicators**

Noise levels can be measured and reported with different indicators, each capturing various aspects of noise exposure. The 2018 Environmental Noise Guidelines for the WHO European Region^4^ provide a detailed description of all indicators, as outlined in Table S2:

| **Noise indicator** | **Description** |
| --- | --- |
| L_Aeq,T_ | A-weighted, equivalent continuous sound pressure level during a stated time interval starting at t1 and ending at t2, expressed in decibels (dB), at a given point in space. |
| L_A,max_ | Maximum time-weighted and A-weighted sound pressure level within a stated time interval starting at t1 and ending at t2, expressed in dB. |
| L_AF_ | A-weighted sound pressure level with FAST time constant as specified in IEC 61672-11. |
| L_AF,max_ | Maximum time-weighted and A-weighted sound pressure level with FAST time constant within a stated time interval starting at t1 and ending at t2, expressed in dB. |
| L_AS,max_ | Maximum time-weighted and A-weighted sound pressure level with SLOW time constant within a stated time interval starting at t1 and ending at t2, expressed in dB. |
| L_E_ | Sound energy density level is the logarithmic ratio of the time-averaged sound energy per unit volume to the reference sound energy density Eo = 10-12 J/m3. |
| L_ex,8h_ | L_eq_ (equivalent continuous sound level) corrected for the length of the working shift, in this case 8 hours. |
| L_day_ | Equivalent continuous sound pressure level when the reference time interval is the day. |
| L_den_ | Day-evening-night-weighted sound pressure level as defined in section 3.6.4 of ISO 1996-1:2016. |
| L_dn:_ | Day-night-weighted sound pressure level as defined in section 3.6.4 of ISO 1996-1:2016. |
| L_evening_ | Equivalent continuous sound pressure level when the reference time interval is the evening. |
| L_night_ | Equivalent continuous sound pressure level when the reference time interval is the night. |
| L_peak,C_ | Level of peak sound pressure with C-weighting, within a specified time interval. |
| L_peak,lin_ | Level of peak sound pressure with linear frequency weighting, within a specified time interval. |

**1.3. Table S3: Definitions of data extraction items**

Study characteristics of all studies were extracted using the Global Burden of Disease (GBD) standardized data extraction template.

| **Category** | **Variable** | **Definition** |
| --- | --- | --- |
| Source | seq | For RR bundle upload: A null value in the seq column indicates new data that should be inserted into the database [leave blank]. |
|  | underlying_nid (unique study identifier) | Underlying NID: Enter the underlying NID of the study (if applicable). Always talk to a data indexer if you don't know if an underlying NID is needed. They may be used for meta-analyses, certain database sources, and in some other specific cases. |
|  | nid (unique study identifier) | Found in Global Health Data Exchange (GHDx), created through the epi form, or created by Data Indexer. |
|  | field_citation_value | IHME Zotero format or if source has NID, citation info from GHDx. |
|  | file_path | optional; full file path of article; Only needed if source doesn't have NID, to facilitate NID creation. |
| Risk-Outcome pair | rei | Risk: Select the risk factor, if not listed here, contact the causal criteria team. |
|  | acause | Outcome: Select the outcome. |
| Study Design | design | Study design: Specify the design of the study. Choose 1. |
|  | source_type | Source type: Specify source type of study. Choose 1. |
|  | study | Study Name: Enter the name of the study (e.g., Nurses' Health Study), if provided. Do not enter the title of the article. |
| Location | location_name | location name (from locations tab). There is a dropdown menu, but it may be easier to reference to locations tab and search for your location. |
|  | location_id | autopopulated from location_name. |
|  | smaller_site_unit | 1 = if data are collected in a location more granular than the assigned location_id=1; 0 = data were not collected in a location more granular than the assigned location_id. |
|  | site_memo | open-text site field; direct copy and paste from article of all information regarding location of data collection. |
| Study Population | year_start | year the study was started..If article does not specify year_start and/or year_end, the general rule is to use the default of year_start = (PubYear - 3) and year_end = (PubYear - 1). Always provide (brief) documentation in note_SR if you have to make assumptions about these fields (i.e., 'Years of data collection not specified; used PubYear - 3; PubYear - 1.'. If information in the article makes the default seem inappropriate (e.g., the year of acceptance of the article is 2 years before the publication year), you can modify the default. Always document why you used the years you entered if you are unsure. |
|  | year_end | year the study was finished (including most recent follow up). If article does not specify year_start and/or year_end, the general rule is to use the default of year_start = (PubYear - 3) and year_end = (PubYear - 1). Always provide (brief) documentation in note_SR if you have to make assumptions about these fields (i.e., 'Years of data collection not specified; used PubYear - 3; PubYear - 1.'. If information in the article makes the default seem inappropriate (e.g., the year of acceptance of the article is 2 years before the publication year), you can modify the default. Always document why you used the years you entered. |
|  | age_start | ages from 1 and above must be entered as an integer. Ages <1 can be entered as decimal values, e.g., 3 days = 3/365. |
|  | age_end | ages from 1 and above must be entered as an integer. Ages <1 can be entered as decimal values, e.g., 3 days = 3/365. |
|  | age_issue | 0 = no issue flagged; 1 = issue flagged for modeler; always include explanatory notes the note_SR column. |
|  | percent_male | what percent of the population is male (0-1), if pop is all female then it would be 0. |
|  | sex | Specify if the study population is exclusively male, female or both? |
|  | sex_issue | Mark this column with a 1 to flag that there is an issue related to the sex column. |
|  | rep_geography | Were the study participants representative of the geography? 1=yes, 0=no. |
|  | rep_selection_criteria | If rep_geography is no, please specify the selection criteria of the study. |
|  | rep_prevalent_disease | Have all the study participants developed the outcomes of interest, and does the study aim to measure outcome of exposure? 1=yes 0=no (i.e. yes if for SBP-IHD paper, partcipants have IHD and the paper is looking at mortality due to SBP, no if for SBP-IHD paper the participants have other prevalent diseases). |
| Exposure | exp_def | Exposure definition: Provide a brief description of the exposure definition as reported in the study (e.g., smoking tobacco products over the past year). |
|  | exp_timing | Timing of exposure assessment: Please specify the timing of the exposure assessment with repect to occurrence of the disease endpoint. |
|  | exp_baseline | Was the exposure assessed only at baseline? |
|  | exp_fup_num | Specify the number of times that exposure was assessed excluding baseline. |
|  | exp_type | Which form of the exposure was included in relative risk estimation analysis? |
|  | exp_level | Level of exposure assessment: The exposure was assessed… |
|  | exp_method_1 | Please specify the method of exposure assessment. If there are more than 1, please add in the next columns labeled "exp_method_2". |
|  | exp_method_2 | Please specify the method of exposure assessment. If there are more than 2, please add in the next columns labeled "exp_method_3". |
|  | exp_method_3 | Please specify the method of exposure assessment. |
|  | exp_recall_type | This field describes the unit of exposure recall used in data collection. Select the correct option from the drop-down menu. If the unit is days, weeks, months, or years, please enter the number in exp_recall_type_value (next column). If the unit is 'lifetime', nothing needs to be entered in exp_recall_type_value. For example, if the study said the recall period was 4 weeks, enter 4 in exp_recall_type_value, and 'weeks' in the field exp_recall_type. If 'other' is selected, please describe in exp_recall_type_other. |
|  | exp_recall_type_value | If you entered days, weeks, months, or years in the field 'exp_recall_type', please enter the corresponding integer in this field. For example, if the study said the recall period was 4 weeks, enter 4 in exp_recall_type_value, and 'weeks' in the field exp_recall_type. |
|  | exp_recall_type_other | If 'other' was selected in exp_recall_type, please describe the exposure recall period that the study specified (e.g., recall of exposure from 12 - 18 years). |
|  | exp_meas_num | If the exposure level was assessed multiple times at a given time point (e.g., systolic blood pressure), specify the number of measurements at each time point. |
|  | exp_biomarker | If the exposure level was assessed via a biomarker, specify the full name of the biomarker. |
|  | exp_kilometer | Specify the geographical unit of measurement in kilometer (if applicable, e.g., satellite data). |
|  | exp_instrument | Exposure assessment instrument: Specify the name of the exposure assessment instrument. For self-reported exposures, please specify the name of the questionnaire e.g., International Physical Activity Questionnaire (IPAQ). |
| Outcome | outcome_def | Outcome definition: Provide a brief description of the outcome as reported in the study. |
|  | outcome_type | Outcome type: please specify if the outcome definition included incidence of or mortality from a disease endpoint. |
|  | outcome_assess_1 | Method of outcome assessment: Specify the method of assessment of the study outcome. If more than 1 are appropriate, enter additional methods in the next column labeled "outcome_assess_2". |
|  | outcome_assess_2 | Method of outcome assessment: Specify the method of assessment of the study outcome. If more than 1 are appropriate, enter additional methods in the next column labeled "outcome_assess_3". |
|  | outcome_assess_3 | Method of outcome assessment: Specify the method of assessment of the study outcome. |
| Follow up | duration_fup_units | Units of follow up duration. |
|  | value_of_duration_fup | Enter the average length of participant follow-up. |
|  | dropout_assess | Specify how dropout rate was defined in the study. |
|  | dropout_rate | Dropout rate: Specify the dropout rate (%) at the end of the study. Enter on a "per 1" basis. For example: 23% is entered as .23. |
| Effect Size | page_num_effect_size | Page number (where you found effect_size) from literature, or survey question where you found effect size; Use page number(s) of article, not page # of pdf. |
|  | effect_size_measure | Effect size measure: Specify the measure of effect size. Select 1. |
|  | effect_size | Effect size estimate: Provide the effect size estimate. This can also be thought of as your measure. In situations where there are 2 models (a basic and an adjusted) and the only difference are the confounders that were adjusted for, the preference will always be towards the more adjusted model. Extractors may extract the basic model as well if they deem it necessary/helpful but should indicate that this is duplicate data. |
|  | effect_size_unit | Specify effect size unit. Choose 1. |
|  | lower | Provide the lower limit of the confidence interval. Enter on a "per 1" basis. (If the CI is reported as a percent, you must convert to a decimal.) These 3 fields must all be filled in if any of them are filled in: lower, upper, uncertainty_type_value. |
|  | upper | Provide the upper limit of the confidence interval. Enter on a "per 1" basis. (If the CI is reported as a percent, you must convert to a decimal.) These 3 fields must all be filled in if any of them are filled in: lower, upper, uncertainty_type_value. |
|  | CI_uncertainty_type_value | This field is required if 'lower' & 'upper' are entered. This column represents the confidence level which is reported at (Eg. 95, 90, 99). These 3 fields must all be filled in if any of them are filled in: lower, upper, uncertainty_type_value. |
|  | nonCI_uncertainty_value | Numerical value of the nonCI_uncertainty_type entered in that column. For example, if SD=5.3, you'd put 5.3 in this column, and choose SD from the drop down menu in nonCI_uncertainty_type. |
|  | nonCI_uncertainty_type | Enter SE or SD if appropriate. For example, if SD=5.3, you'd put 5.3 in nonCI_uncertainty_value, and choose SD from the drop down menu in this column (nonCI_uncertainty_type). |
|  | effect_size_multi_location | 1 if the reported effect size is from a multi-country study and only one ffect size has been reported for all locations, otherwise 0. |
|  | pooled_cohort | 1 if the reported effect size is from a pooled analysis and only pooled effect size has been reported, otherwise 0. |
|  | dose_response | Does the study support a dose-response relationship between the exposure and the outcome?  External Collaborators: This field is not relevant to all study types so please judge accordingly. |
|  | dose_response_detail | If 1 was specified in the dose_response field, please specify in this field the type of evidence supporting the dose-response relationship. For example, "statistically significant p value for linear trend". |
| Confounders | confounders_age | if included in the relative risk estimation analysis, mark 1 for yes. Mark 0 for no. |
|  | confounders_sex | if included in the relative risk estimation analysis, mark 1 for yes. Mark 0 for no. |
|  | confounders_education | if included in the relative risk estimation analysis, mark 1 for yes. Mark 0 for no. |
|  | confounders_income | if included in the relative risk estimation analysis, mark 1 for yes. Mark 0 for no. |
|  | confounders_smoking | if included in the relative risk estimation analysis, mark 1 for yes. Mark 0 for no. If you are unsure whether this confounder needs to be accounted for, please talk to your modeler or IHME point of contact (if you are an external collaborator). |
|  | confounders_alcohol_use | if included in the relative risk estimation analysis, mark 1 for yes. Mark 0 for no. If you are unsure whether this confounder needs to be accounted for, please talk to your modeler or IHME point of contact (if you are an external collaborator). |
|  | confounders_physical_activity | if included in the relative risk estimation analysis, mark 1 for yes. Mark 0 for no. If you are unsure whether this confounder needs to be accounted for, please talk to your modeler or IHME point of contact (if you are an external collaborator). |
|  | confounders_dietary_components | if included in the relative risk estimation analysis, mark 1 for yes. Mark 0 for no. If you are unsure whether this confounder needs to be accounted for, please talk to your modeler or IHME point of contact (if you are an external collaborator). |
|  | confounders_bmi | if included in the relative risk estimation analysis, mark 1 for yes. Mark 0 for no. If you are unsure whether this confounder needs to be accounted for, please talk to your modeler or IHME point of contact (if you are an external collaborator). |
|  | confounders_hypertension | if included in the relative risk estimation analysis, mark 1 for yes. Mark 0 for no. If you are unsure whether this confounder needs to be accounted for, please talk to your modeler or IHME point of contact (if you are an external collaborator). |
|  | confounders_diabetes | if included in the relative risk estimation analysis, mark 1 for yes. Mark 0 for no. If you are unsure whether this confounder needs to be accounted for, please talk to your modeler or IHME point of contact (if you are an external collaborator). |
|  | confounders_hypercholesterolemia | if included in the relative risk estimation analysis, mark 1 for yes. Mark 0 for no. If you are unsure whether this confounder needs to be accounted for, please talk to your modeler or IHME point of contact (if you are an external collaborator). |
|  | confounders_other | Use this field to list other confounders that were not mentioned in previous columns that were adjusted for in the analysis. |
| Cohorts | cohort_person_years_exp | Please specify the person years of follow up in the exposed group. |
|  | cohort_person_years_unexp | Please specify the person years of follow up in the unexposed group. |
|  | cohort_person_years_total | Enter the total person-years of follow-up if person-years of follow up in exposed and unexposed not reported. |
|  | cohort_number_events_exp | Please specify the number of events in the exposed group. |
|  | cohort_number_events_unexp | Please specify the number of events in the unexposed group. |
|  | cohort_number_events_total | Enter the total number of events/cases if number of events in exposed and unexposed not reported. |
|  | cohort_sample_size_exp | Please specify the number of people in the exposed group if person-years of follow up in exposed not reported. |
|  | cohort_sample_size_unexp | Please specify the number of people in the unexposed group if person-years of follow up in unexposed not reported. |
|  | cohort_sample_size_total | Please specify the number of people included in the analysis if total person-years of follow up in not reported. |
|  | cohort_exposed_def | Exposed group definition: Provide a brief description of the exposed group for which the relative risk is reported (e.g., current smokers). |
|  | cohort_unexposed_def | Unexposed group definition: Provide a brief description of the unexposed group (i.e., the comparison group) as used in estimation of the relative risk (e.g., never smokers). |
|  | cohort_exp_unit_rr | Exposure unit (for continuous risks): Specify the unit of exposure (e.g., grams/day). |
|  | cohort_exp_level_rr | Exposure level in the exposed group (for continuous risks): Specify the mean/median level of exposure in the exposed group. |
|  | cohort_unexp_level_rr | Exposure level in the unexposed group (for continuous risks): Specify the mean/median level of exposure in the unexposed group. |
|  | cohort_exp_level_dr | Exposure level in for dose-repose RRs (for continuous risks): If the study reports dose-repose RR, please specify the level of exposure for the reported RR. |
| Case-control | cc_community | Were the controls selected from the community? 1 = yes, 0=no. |
|  | cc_cases | Number of cases. |
|  | cc_control | Number of controls. |
|  | cc_exposed_def | Exposed group definition: Provide a brief description of the exposed group for which the relative risk is reported (e.g., current smokers). |
|  | cc_unexposed_def | Unexposed group definition: Provide a brief description of the unexposed group (i.e., the comparison group) as used in estimation of the relative risk (e.g., never smokers). |
|  | cc_exp_unit_rr | Exposure unit (for continuous risks): Specify the unit of exposure (e.g., grams/day). |
|  | cc_exp_level_rr | Exposure level in the exposed group (for continuous risks): Specify the mean/median level of exposure in the exposed group. |
|  | cc_unexp_level_rr | Exposure level in the unexposed group (for continuous risks): Specify the mean/median level of exposure in the unexposed group. |
|  | cc_exp_level_dr | Exposure level in for dose-repose RRs (for continuous risks): If the study reports dose-repose RR, please specify the level of exposure for the reported RR. |
| Covariates | cov_subpopulation | 0 if generalizable--general population with reasonable exclusions, 1 analysis in subgroup such as high_risk group. |
|  | cov_exposure_population | 0 for individual and 1 for population level exposure. (For Air Pollution Risks, we consider <1km to be individual). |
|  | cov_exposure_selfreport | 0 for measured exposure/objective measurement and 1 for self report. |
|  | cov_exposure_study | 0 for exposure measured multiple times and 1 for only at baseline. |
|  | cov_outcome_selfreport | 0 outcome based on death certificate or medical record and 1 if self-report. |
|  | cov_outcome_unblinded | 0 if assessment of outcome is blind to exposure or vise versa and 1 if unblinded. |
|  | cov_reverse_causation | 0 no risk of reverse causation and 1 if there is a risk. |
|  | cov_confounding_nonrandom | 0 for randomized study and 1 for non randomized. |
|  | cov_confounding_uncontrolled | 0 for randomization or outcome controlled for all major known confounders and age, sex, education, income, and all critical determinants of outcome (this varies depending on the risk). 1 for age, sex, and other critical determinants. 2 for only age and sex. |
|  | notes_confounders | Please specify all confounders controlled for in analysis. |
|  | cov_selection_bias | 0 for greater than 95% follow-up, 1 for follow up of 85-95%, and 2 for less than 85% follow up. Case-control studies should be scored based on the percentages of cases and controls for which exposure data could be ascertained (avg of int/control or higher bin) *Note: this may not be the best way to capture selection bias in non RCT studies so consider creating your own true selection bias covariate if recognized as something needed. |
|  | cov_mixed_intervention | 0 if the intervention only has to with your risk of focus, 1 if the intervention includes multiple risks. |
|  | notes_mixed_intervention | Optional: If cv_mixed_interventions = 1, you can use this column to specify other risks in intervention. |
|  | cov_exp_measurement | Optional (use if its helpful): multiple prospective measurements (0) vs single baseline prospective measurement (1) vs retrospective measurement/case-control (2). |
| Other | note_modeler | for modelers only, audience is modeler, not for correspondence. |
|  | note_sr | notes related to extraction, including assumptions, data adjustment, problems with source, any other notes that may be relevant, etc. |
|  | extractor | uwnet id of person who extracted the data. |
|  | input_type | specify input type. Choose 1. Use input_type tab if drop down is hard to read. This field in most ocassions should be "extracted". |
|  | SES characteristics | If 'education' or 'income' were controlled in analysis (confounders) specify in 'Notes' any data/estimates. |
|  | Correlated exposures | specify if correlated exposures are taken into account. Choose 1 if yes. If so, mention in 'Notes' which exposures. Choose 0 if no. |

**1.4 Table S4: Standard bias covariates**

| **Bias category** | **Bias covariate name** | **Description** | **Values** |  |
| --- | --- | --- | --- | --- |
| Representativeness of study population | Representativeness | Study sample is not geographically representative of underlying location. | 0 if no; 1 if yes |  |
|  | cov_subpopulation | Study sample represents a sub-group of population, e.g. only males/females. | 0 if no; 1 if yes |  |
| Selection bias | cov_selection_bias | Captures if study is at risk for selection bias. Study determined to be at risk for selection bias if loss to follow-up (cohorts) or percent for whom data not ascertained (case-controls) > 20%. | 0 if no; 1 if yes |  |
|  |  |  |  |  |
| Reverse causation | cov_reverse_causation | Captures if study is at risk for reverse causation. Case-control studies are assumed to be at risk of reverse-causation. | 0 if no; 1 if yes |  |
|  |  |  |  |  |
| Control for confounding | cov_confounding_uncontrolled | Captures if a study reports completely un-adjusted estimates of association (raw or crude effects sizes). | 0 if no; 1 if yes |  |
|  |  |  |  |  |
| Correlated exposure | correlated_exposure | Specify if correlated exposures are taken into account in the fully adjusted model. | 0 if no; 1 if yes |  |
|  |  |  |  |  |
| Other | hazard_ratio | Study reports and odds ratio (reference: relative risk). | 0 if no; 1 if yes |  |

**1.5 Exposure Assessment and Assignment**

Exposure to transportation noise sources (i.e. road traffic, railway, and aircraft) can be reported based on predictive modelling or physical measurement. Any exposure assessment was considered in this review, if it concerned long-term exposure (i.e. a year or multiple years).^5^

- Predictive models: prediction methods that allow to predict noise given a series of traffic parameters and topographical parameters are known. The most common method is the Nordic prediction method, which consists in three different steps, namely: (1) Development of noise emissions; (2) Modelling dispersion and quantification of transmission at the façade; and (3) Considering sound insulation. Several parameters can be considered and will make the predictions more complex and more reliable, but the necessary input data are emissions, dispersion and characteristics of the receiver.
- Physical measurement: a grid map of the city of interest is defined and investigators repeat measurement in each of those streets to average results and define noise exposure for each of the residential addresses that are associated to that specific street. Different protocols have been available; few studies have adopted this approach, mostly because of the effort it requires and because results of predictions models are more accurate compared to physical measurement.

**Section 2: Meta-analysis: supplementary methods**

**2.1 Data pre-processing**

Different environmental noise exposure metrics are used to measure transportation noise (e.g. L_eq24h_, L_day_, L_evening_, L_night_, L_dn_, and L_den_). However, pooling measures of risk requires re-scaling to a common metric of exposure. Rules to perform re-scaling were derived empirically using two methods: i) analysis of diurnal variation of traffic. ii) analysis of differences between calculated noise exposure metrics.^6^ Values obtained to re-scale from one metric to another vary on the source of transportation noise, namely:

- Road traffic noise:
  - L_DEN_ = L_AEQ24_ + 3.6
  - L_DEN_ = L_AEQ16_ + 2.4
  - L_DEN_ = L_NIGHT_ + 8.3
- Railway noise:
  - L_DEN_ = L_AEQ24_ + 6.1
  - L_DEN_ = L_AEQ16_ + 6
  - L_DEN_ = L_NIGHT_ + 6.6
- Aircraft noise:
  - L_DEN_ = L_AEQ24_ + 3.6
  - L_DEN_ = L_AEQ16_ + 2.4
  - L_DEN_ = L_NIGHT_ + 10.1

After re-scaling the upper and lower limit of exposure into the common metric of referral, the midpoint of the exposure range was used to estimate the linear trend of exposure and were modeled against the associated risk measure using the *dosresmeta* function.^7^ This allowed to re-scale all measures or relative risk to 10 dB increment, to make them comparable and suitable to perform conventional meta-analysis.

**2.2. Conventional Meta-Analysis: methods**

Meta-analysis is a statistical technique used to aggregate estimates of association for a risk-outcome pair, derived from multiple studies.^8^ Following a systematic literature review, estimates of association and their corresponding confidence interval are extracted from the included studies, and are used to compute the respective beta coefficient and standard error. Depending on the degree of heterogeneity among the estimates, which can be tested using Cochran’s Q test^9^, a fixed or random effects model^10^ can be chosen. Moreover, homogeneity can be assessed with I^2^ index and provides an estimate of the percentage that is due to real differences between studies and not due to chance.^11^ The pooled estimate will be obtained as shown in equation [1]:

$$\theta_{pooled}=\frac{\sum_{i} w_{i}\theta_{i}}{\sum_{i} w_{i}}$$

[1]

Where, for a fixed effect model, the weights are calculated with the inverse variance method as in [2]

$$w_{i}=\frac{1}{\sigma_{i}^{2}}$$

[2]

On the other hand, for a random effect model, weights are obtained as [3] shows

$$w_{i}=\frac{1}{\sigma_{i}^{2}+\tau^{2}}$$

[3]

Where $\tau^{2}$ is the variance between studies, which considers the heterogeneity found between the different estimates. An important phase of a meta-analysis is evaluating presence of reporting and publication bias, which can be assessed with Egger’s test, funnel plot and more other tests.^12,13,14^

**Section 3: Systematic review: supplementary results**

3.1 Table S5: Characteristics of included studies

| **Author** | **Year** | **Country** | **Source of transportation noise** | **Disease outcome** | **Study design and Years** | **Exposure Assessment and assignment** | **Metric** | **Variables adjusted*** | **Quality**  **assessment**** |
| --- | --- | --- | --- | --- | --- | --- | --- | --- | --- |
| Andersson *et al.*^15^ | 2020 | Sweden | Road traffic | IHD, Stroke | Prospective (1970-2011) | Predictive models (Household) | Incidence, Mortality | 1-5,7,9,11-13,18,20,62 | High |
| Babisch *et al.* ^16^ | 1994 | Germany | Road traffic | MI | Case-control (1994) | Physical measurement (Household) | Incidence | 1,2,4,9,23,  29,57 | Medium |
| Babisch *et al.* ^17^ | 1999 | United Kingdom | Road traffic | IS | Prospective (1979-1993) | Physical measurement (Household) | Incidence | 1,2,4,7,9,23,  26,29,59,61,62 | Medium |
| Babisch *et al.* ^18^ | 2005 | Germany | Road traffic | MI | Case-control (1998-2001) | Physical measurement (Household) | Incidence | 1-4,9-11,26,29,41,47,  48,58,60 | Medium |
| Bai *et al.* ^19^ | 2020 | Canada | Road traffic | MI | Retrospective (2001-2015) | Predictive models (Household) | Incidence | 1,2,3,5,10,  11,61 | High |
| Bodin *et al.* ^20^ | 2016 | Sweden | Road traffic | MI | Prospective (2000-2010) | Predictive models (Household) | Incidence | 1-4,6,7,9,13,  14,62 | Medium |
| Carey *et al.* ^21^ | 2016 | United Kingdom | Road traffic | MI, IS, Stroke | Prospective (2005-2011) | Predictive models (Population) | Incidence | 1,2,4,9,15 | High |
| Cole‑Hunter *et al.* ^22^ | 2021 | Denmark | Road traffic | IS, HS, Stroke | Prospective (1993-2014) | Predictive models (Household) | Incidence | 1,2,4,6,7,13,  16,17,62 | High |
| Dimakopoulou *et al.*^23^ | 2017 | Greece | Aircraft and road traffic | MI, Stroke | Prospective (2004-2013) | Predictive models (Household) | Incidence | 1-4,6,7,9,18 | Medium |
| Grady *et al.* ^24^ | 2023 | United States of America | Aircraft noise | CVDs | Prospective (1994-2014) | Predictive models (Household) | Incidence, Mortality | 1,2,4,6,7,8,  17,21-27,62 | High |
| Hao *et al.* ^25^ | 2022 | United Kingdom | Road traffic | IHD, IS, MI, Stroke, | Prospective (2006-2021) | Predictive models (Household) | Incidence, Mortality | 1-4,6-8,10-12,17,21,  26,28 | High |
| Lim *et al.* ^26^ | 2021 | Denmark | Road traffic | MI | Prospective (1993-2014) | Predictive models (Household) | Incidence, Mortality | 1,2,4,6,7,9,  13,29-33,63 | High |
| Magnoni *et al.* ^27^ | 2021 | Italy | Road traffic | MI, IS, Stroke | Retrospective (2001-2018) | Predictive models (Household) | Incidence | 1,2,17,34-37 | High |
| Poulsen *et al.* ^28^ | 2023 | Denmark | Road traffic | Stroke | Prospective (2005-2017) | Predictive models (Household) | Incidence | 1,2,3,5,13,  14,29,38,62 | High |
| Pyko *et al.* ^29^ | 2019 | Sweden | Aircraft, railway, and road traffic | IS, Stroke | Prospective (1990-2010) | Predictive models (Household) | Incidence | 1-4,6,7,13,  29,39,62 | High |
| Roswall *et al.* ^30^ | 2017 | Denmark | Road traffic | MI | Prospective (1997-2011) | Predictive models (Household) | Incidence, Mortality | 1-4,6,7,8,  13,40,41 | High |
| Seidler *et al.* ^31^ | 2016 | Germany | Aircraft, railway, and road traffic | IS, HS, Stroke | Case-control (2005-2010) | Physical measurement (Household) | Incidence, Mortality | 1,2,3,29,42 | High |
| Seidler *et al.* ^32^ | 2018 | Germany | Aircraft, railway, and road traffic | MI | Case-control (2005-2010) | Physical measurement (Household) | Incidence | 1,2,3,43,44 | High |
| Selander *et al.* ^33^ | 2009 | Sweden | Road traffic | Stroke | Case-control (1992-1994) | Predictive models (Household) | Incidence, Mortality | 1,2,4,7,11,  45-47 | High |
| Sørensen *et al.* ^34^ | 2012 | Denmark | Railway and road traffic | IS, HS, Stroke | Prospective (1993-2006) | Predictive models (Household) | Incidence | 1-9,13,41,  48,49,50 | High |
| Sørensen *et al.* ^35^ | 2014 | Denmark | Road traffic | IS | Prospective (1997-2009) | Predictive models (Household) | Incidence | 1-4,6-9,13,  50,51 | Medium |
| Sørensen *et al.* ^36^ | 2021 | Denmark | Road traffic | MI | Prospective (2000-2017) | Predictive models (Household) | Incidence | 1,2,3,5,13,  29,41,52-55,62 | High |
| Thacher *et al.* ^37^ | 2022 | Denmark | Aircraft, railway, and road traffic | IHD, MI | Prospective (2005-2017) | Predictive models (Household) | Incidence | 1,2,3,5,13,14,  29,36,38,62 | High |
| Vienneau *et al.* ^38^ | 2022 | Switzerland | Aircraft, railway, and road traffic | HS, IHD, IS, MI, Stroke | Prospective (2001-2015) | Predictive models (Household) | Incidence | 1,2,3,13,17,  23,29,37,62,64 | High |
| Willich *et al.* ^39^ | 2005 | Germany | Road traffic | MI | Case-control (1998-2001) | Physical measurement (Household) | Incidence | 1-4,9-12,26,29,  56-58 | Medium |
| Yankoty *et al.* ^40^ | 2021 | Canada | Road traffic | MI | Retrospective (2000-2014) | Predictive models (Household) | Incidence | 1,2,3,5,49 | High |
| * 1: Age; 2: Sex; 3: Education; 4: Smoking; 5: Income; 6: Alcohol Use, 7: Physical Activity; 8: Dietary Components; 9: BMI; 10: Hypertension; 11: Diabetes; 12: Hypercholesterolemia; 13: Calendar year of cohort entry; 14: Country of birth; 15: Index of multiple deprivation (Income deprivation, employment deprivation, health deprivation and disability, education deprivation, crime deprivation, barriers to housing and services deprivation, living environment deprivation); 16: Fruit Consumption; 17: PM_2.5_; 18: Salt intake; 19: Stress; 20: Heredity; 21: Ethnicity; 22: Spouse’s education attainment; 23: nSES score (socio-economic status); 24: Region of residence; 25: Population density; 26: Family history; 27: Menopausal status; 28: Sleep duration; 29: Employment status; 30: Use of oral contraceptive; 31: Hormone replacement therapy; 32: Level of urbanization; 33: Parity; 34: Italian deprivation index (percentage of population with poor education; unemployment; occupied dwellings for rent; percentage of single parents with dependent children; population density); 35: NO_2_; 36: Surrounding greenness; 37: Citizenship; 38: Neighborhood (Population with low income, with only basic education, who are unemployed, with manual labor, with non-Western background, with criminal record, who are sole-provider, who live in social housing); 39: Year of the event; 40: Socio-economic level area; 41: Railway noise exposure; 42: SGB II-rate (proportion of persons entitled to SGB II benefits (LB) relative to the population under 65 years); 43: Job title; 44: Local proportion of people receiving unemployment benefits; 45: Catchment area; 46: Air pollution; 47: Occupational noise exposure; 48; Aircraft Noise; 49: NOx; 50: Smoking intensity; 51: Municipality SES; 52: Region of origin; 53: Manual labor; 54: Single parent; 55: Criminal record; 56: Number of working hours; 57: Shift work; 58: Living alone; 59: Prevalence of IHD; 60: Noise sensitivity score; 61: Preexisting comorbidities (such as stroke, asthma, chronic obstructive pulmonary diseases and cancer); 62: Civil status; 63: Job strain; 64: Mother tongue  **Blue corresponds to studies scoring between 7-9 points on the Newcastle-Ottawa Scale instrument and classified as high quality. Light-blue corresponds to studies scoring between 4-6 points on the Newcastle-Ottawa Scale instrument and classified as medium quality.^41^ | | | | | | | | | |

**3.1.1 Figure S1: Number of included studies by geographical location**

The lowest number of studies was conducted in the US (n=1) and Canada (n=2), whereas the highest number of studies was conducted in Denmark (n=8), followed by Germany (n=5).

**3.1.2 Figure S2: Number of identified risk-outcome pairs**

The risk-outcome pair that was studied the most was road traffic noise and myocardial infarction (n=16), followed by road traffic noise and stroke (n=11). The risk-outcome pairs involving railway (n=3) and aircraft noise (n=4) were the least studied.

**Section 4: Meta-analysis: supplementary results**

**4.1. Supplementary results for conventional meta-analysis**

We present supplementary results from conventional meta-analysis, stratified by type of transportation noise source (i.e. road traffic, railway, and aircraft noise) and health indicators (i.e. non-fatal *versus* fatal). We focused on risk estimates derived from *fully* adjusted models that account for air pollutants. We present results only for risk–outcome pairs with sufficient data to permit meta-analysis. Studies were deemed appropriate for meta-analysis if at least three independent studies reported the same or equivalent effect statistics, and if they had an equivalent exposure, disease outcome, and health indicators, thereby enabling meaningful and interpretable pooling of estimates.

4.1.1. **Road Traffic Noise**
       4.1.1.1. **Myocardial infarction: non-fatal**

The pooled relative risk estimate was 1.04 (95%CI: 0.98-1.11). Between-study heterogeneity was high (I^2^=87.7%, p-value < 0.0001).

**Figure S3:** Forest plot for road noise and non-fatal myocardial infraction

4.1.1.2. **Myocardial infarction: fatal**

The pooled relative risk for fatal myocardial infarction was 1.04 (95%CI: 1.03-1.05), indicating a 4% increased risk per 10 dB increment. Between study heterogeneity was moderate (I^2^=37.3%, p-value=0.19)

**Figure S4:** Forest plot for road noise and fatal myocardial infraction

4.1.1.3. **Ischemic Heart Disease: non-fatal**

The pooled relative risk for the association between road noise and non-fatal IHD was equal to 1.03 (95%CI: 0.90-1.18), indicating no association, with high heterogeneity (I^2^=90.2%, p-value < 0.0001).

**Figure S5:** Forest plot for road noise and non-fatal ischemic heart disease

4.1.1.4. **Stroke: non-fatal**

The pooled relative risk for the association between road noise and non-fatal stroke was equal to 1.03 (95%CI: 1.02-1.04), indicating 3% increase of risk, per 10 dB increment in noise exposure. Moderate heterogeneity was observed (I^2^=38%, p-value=0.07).

**Figure S6:** Forest plot for road noise and non-fatal stroke

4.1.1.5. **Stroke: fatal**

The pooled relative risk for the association between road noise and fatal stroke was equal to 1.05 (95%CI: 0.94-1.17), indicating no association, with high heterogeneity (I^2^=95.2%, p-value < 0.0001).

**Figure S7:** Forest plot for road noise and fatal stroke

4.1.2. **Railway Noise**
       4.1.2.1. **Stroke: non-fatal**

The pooled relative risk was equal to 1.00 (95%CI: 0.92-1.07), indicating no association, with high heterogeneity (I^2^=85%, p-value < 0.01).

**Figure S8:** Forest plot for railway noise and non-fatal stroke

4.1.3. **Aircraft Noise**
       4.1.3.1. **Stroke: non-fatal**

The pooled relative risk for the association between aircraft noise and non-fatal stroke was equal to 0.98 (95%CI: 0.95-1.00), indicating no association, with absence of heterogeneity (I^2^=0%, p-value = 0.97).

**Figure S9:** Forest plot for aircraft noise and non-fatal stroke

**Section 5: Research and reporting practices**

**5.1** **Table S6: PRISMA 2020 checklist**

| **Section and Topic** | **Item #** | **Checklist item** | **Location where item is reported** | |  |
| --- | --- | --- | --- | --- | --- |
| **TITLE** | | | |  | |
| Title | 1 | Identify the report as a systematic review. | Page 1 | |  |
| **ABSTRACT** | | | |  | |
| Abstract | 2 | See the PRISMA 2020 for Abstracts checklist. | Page 2 | |  |
| **INTRODUCTION** | | | |  | |
| Rationale | 3 | Describe the rationale for the review in the context of existing knowledge. | Pages 3,4 | |  |
| Objectives | 4 | Provide an explicit statement of the objective(s) or question(s) the review addresses. | Page 3 | |  |
| **METHODS** | | | |  | |
| Eligibility criteria | 5 | Specify the inclusion and exclusion criteria for the review and how studies were grouped for the syntheses. | Pages 3,4 | |  |
| Information sources | 6 | Specify all databases, registers, websites, organisations, reference lists and other sources searched or consulted to identify studies. Specify the date when each source was last searched or consulted. | Pages 3,4 | |  |
| Search strategy | 7 | Present the full search strategies for all databases, registers and websites, including any filters and limits used. | Pages 3,4 | |  |
| Selection process | 8 | Specify the methods used to decide whether a study met the inclusion criteria of the review, including how many reviewers screened each record and each report retrieved, whether they worked independently, and if applicable, details of automation tools used in the process. | Pages 3,4 | |  |
| Data collection process | 9 | Specify the methods used to collect data from reports, including how many reviewers collected data from each report, whether they worked independently, any processes for obtaining or confirming data from study investigators, and if applicable, details of automation tools used in the process. | Pages 3,4 | |  |
| Data items | 10a | List and define all outcomes for which data were sought. Specify whether all results that were compatible with each outcome domain in each study were sought (e.g. for all measures, time points, analyses), and if not, the methods used to decide which results to collect. | Pages 3-5 | |  |
|  | 10b | List and define all other variables for which data were sought (e.g. participant and intervention characteristics, funding sources). Describe any assumptions made about any missing or unclear information. | Pages 3-5 | |  |
| Study risk of bias assessment | 11 | Specify the methods used to assess risk of bias in the included studies, including details of the tool(s) used, how many reviewers assessed each study and whether they worked independently, and if applicable, details of automation tools used in the process. | Page 4 | |  |
| Effect measures | 12 | Specify for each outcome the effect measure(s) (e.g. risk ratio, mean difference) used in the synthesis or presentation of results. | Pages 3-5 | |  |
| Synthesis methods | 13a | Describe the processes used to decide which studies were eligible for each synthesis (e.g. tabulating the study intervention characteristics and comparing against the planned groups for each synthesis (item #5)). | Pages 3-5 | |  |
|  | 13b | Describe any methods required to prepare the data for presentation or synthesis, such as handling of missing summary statistics, or data conversions. | Pages 4,5 | |  |
|  | 13c | Describe any methods used to tabulate or visually display results of individual studies and syntheses. | Pages 3-5 | |  |
|  | 13d | Describe any methods used to synthesize results and provide a rationale for the choice(s). If meta-analysis was performed, describe the model(s), method(s) to identify the presence and extent of statistical heterogeneity, and software package(s) used. | Pages 3-5 | |  |
|  | 13e | Describe any methods used to explore possible causes of heterogeneity among study results (e.g. subgroup analysis, meta-regression). | Pages 3-5 | |  |
|  | 13f | Describe any sensitivity analyses conducted to assess robustness of the synthesized results. | Pages 3-5 | |  |
| Reporting bias assessment | 14 | Describe any methods used to assess risk of bias due to missing results in a synthesis (arising from reporting biases). | Page 4 | |  |
| Certainty assessment | 15 | Describe any methods used to assess certainty (or confidence) in the body of evidence for an outcome. | Pages 3-5 | |  |
| **RESULTS** | | | |  | |
| Study selection | 16a | Describe the results of the search and selection process, from the number of records identified in the search to the number of studies included in the review, ideally using a flow diagram. | Pages 5-6 | |  |
|  | 16b | Cite studies that might appear to meet the inclusion criteria, but which were excluded, and explain why they were excluded. | Pages 5-6 | |  |
| Study characteristics | 17 | Cite each included study and present its characteristics. | Pages 5-6 | |  |
| Risk of bias in studies | 18 | Present assessments of risk of bias for each included study. | Page 6 | |  |
| Results of individual studies | 19 | For all outcomes, present, for each study: (a) summary statistics for each group (where appropriate) and (b) an effect estimate and its precision (e.g. confidence/credible interval), ideally using structured tables or plots. | Pages 5-7 | |  |
| Results of syntheses | 20a | For each synthesis, briefly summarise the characteristics and risk of bias among contributing studies. | Pages 5-7 | |  |
|  | 20b | Present results of all statistical syntheses conducted. If meta-analysis was done, present for each the summary estimate and its precision (e.g. confidence/credible interval) and measures of statistical heterogeneity. If comparing groups, describe the direction of the effect. | Pages 5-7 | |  |
|  | 20c | Present results of all investigations of possible causes of heterogeneity among study results. | Pages 5-7 | |  |
|  | 20d | Present results of all sensitivity analyses conducted to assess the robustness of the synthesized results. | Pages 5-7 | |  |
| Reporting biases | 21 | Present assessments of risk of bias due to missing results (arising from reporting biases) for each synthesis assessed. | Pages 5-7 | |  |
| Certainty of evidence | 22 | Present assessments of certainty (or confidence) in the body of evidence for each outcome assessed. | Pages 5-7 | |  |
| **DISCUSSION** | | | |  | |
| Discussion | 23a | Provide a general interpretation of the results in the context of other evidence. | Pages 7-9 | |  |
|  | 23b | Discuss any limitations of the evidence included in the review. | Pages 7-9 | |  |
|  | 23c | Discuss any limitations of the review processes used. | Pages 7-9 | |  |
|  | 23d | Discuss implications of the results for practice, policy, and future research. | Pages 7-9 | |  |
| **OTHER INFORMATION** | | | |  | |
| Registration and protocol | 24a | Provide registration information for the review, including register name and registration number, or state that the review was not registered. | Page 3 | |  |
|  | 24b | Indicate where the review protocol can be accessed, or state that a protocol was not prepared. | Page 3 | |  |
|  | 24c | Describe and explain any amendments to information provided at registration or in the protocol. | Not applicable | |  |
| Support | 25 | Describe sources of financial or non-financial support for the review, and the role of the funders or sponsors in the review. | Page 5 | |  |
| Competing interests | 26 | Declare any competing interests of review authors. | Page 10 | |  |
| Availability of data, code and other materials | 27 | Report which of the following are publicly available and where they can be found: template data collection forms; data extracted from included studies; data used for all analyses; analytic code; any other materials used in the review. | Page 5 | |  |

**5.2 Table S7: Statement of GATHER compliance**

| **Item #** | **Checklist item** | **Reported on page #** |
| --- | --- | --- |
| **Objectives and funding** | | |
| **1** | Define the indicator(s), populations (including age, sex, and geographic entities), and  time period(s) for which estimates were made. | Pages 3-5 |
| **2** | List the funding sources for the work. | Page 5 |
| **Data Inputs** | | |
| *For all data inputs from multiple sources that are synthesized as part of the study:* | | |
| **3** | Describe how the data were identified and how the data were accessed. | Pages 3-5 |
| **4** | Specify the inclusion and exclusion criteria. Identify all ad‐hoc exclusions. | Pages 3-5 |
| **5** | Provide information on all included data sources and their main characteristics. For each data source used, report reference information or contact name/institution, population represented, data collection method, year(s) of data collection, sex and age range,  diagnostic criteria or measurement method, and sample size, as relevant. | Pages 3-5 |
| **6** | Identify and describe any categories of input data that have potentially important biases  (e.g., based on characteristics listed in item 5). | Pages 3-5 |
| *For data inputs that contribute to the analysis but were not synthesized as part of the study:* | | |
| **7** | Describe and give sources for any other data inputs. | Not applicable |
| *For all data inputs:* | | |
| **8** | Provide all data inputs in a file format from which data can be efficiently extracted (e.g., a spreadsheet rather than a PDF), including all relevant meta‐data listed in item 5. For any data inputs that cannot be shared because of ethical or legal reasons, such as third‐party ownership, provide a contact name or the name of the institution that retains the right to  the data. | Pages 4-5 & Appendix |
| **Data analysis** | | |
| **9** | Provide a conceptual overview of the data analysis method. A diagram may be helpful. | Pages 4-5 & Appendix |
| **10** | Provide a detailed description of all steps of the analysis, including mathematical formulae. This description should cover, as relevant, data cleaning, data pre‐processing, data adjustments and weighting of data sources, and mathematical or statistical  model(s). | Pages 4-5 |
| **11** | Describe how candidate models were evaluated and how the final model(s) were  selected. | Pages 4-5 |
| **12** | Provide the results of an evaluation of model performance, if done, as well as the results  of any relevant sensitivity analysis. | Pages 4-5 |
| **13** | Describe methods for calculating uncertainty of the estimates. State which sources of  uncertainty were, and were not, accounted for in the uncertainty analysis. | Pages 4-5 |
| **14** | State how analytic or statistical source code used to generate estimates can be accessed. | Page 5 |
| **Results and Discussion** | | |
| **15** | Provide published estimates in a file format from which data can be efficiently extracted. | Pages 5-7 |
| **16** | Report a quantitative measure of the uncertainty of the estimates (e.g. uncertainty  intervals). | Pages 5-7 |
| **17** | Interpret results in light of existing evidence. If updating a previous set of estimates,  describe the reasons for changes in estimates. | Pages 7-9 |
| **18** | Discuss limitations of the estimates. Include a discussion of any modelling assumptions or  data limitations that affect interpretation of the estimates. | Pages 7-9 |

**Section 6: References**

1. Page MJ, McKenzie JE, Bossuyt PM, Boutron I, Hoffmann TC, Mulrow CD, *et al*. The PRISMA 2020 statement: an updated guideline for reporting systematic reviews. *BMJ* 2021; 372: n71.

2. Stevens GA, Alkema L, Black RE, Boerma JT, Collins GS, Ezzati M, *et al*. Guidelines for Accurate and Transparent Health Estimates Reporting: the GATHER statement. *Lancet* 2016; **388**(10062): e19-e23.

3. GBD 2021 Diseases and Injuries Collaborators. Global incidence, prevalence, years lived with disability (YLDs), disability-adjusted life-years (DALYs), and healthy life expectancy (HALE) for 371 diseases and injuries in 204 countries and territories and 811 subnational locations, 1990–2021: a systematic analysis for the Global Burden of Disease Study 2021. *The Lancet* 2024; **403**(10440): 2133-61.

4. World Health Organization. Regional Office for E. Environmental noise guidelines for the European Region. Copenhagen: World Health Organization. Regional Office for Europe; 2018.

5. Bendtsen H. The Nordic prediction method for road traffic noise. *Science of The Total Environment* 1999; **235**(1): 331-8.

6. Brink M, Schäffer B, Pieren R, Wunderli JM. Conversion between noise exposure indicators Leq24h, LDay, LEvening, LNight, Ldn and Lden: Principles and practical guidance. *Int J Hyg Environ Health* 2018; **221**(1): 54-63.

7. Crippa A, Orsini N. Multivariate Dose-Response Meta-Analysis: The dosresmeta R Package. *Journal of Statistical Software, Code Snippets* 2016; **72**(1): 1-15.

8. Field AP, Gillett R. How to do a meta-analysis. *British Journal of Mathematical and Statistical Psychology* 2010; **63**(3): 665-94.

9. Huedo-Medina TB, Sánchez-Meca J, Marín-Martínez F, Botella J. Assessing heterogeneity in meta-analysis: Q statistic or I² index? *Psychological Methods* 2006; **11**(2): 193-206.

10. Borenstein M, Hedges LV, Higgins JPT, Rothstein HR. A basic introduction to fixed-effect and random-effects models for meta-analysis. *Research Synthesis Methods* 2010; **1**(2): 97-111.

11. Borenstein M, Higgins JPT, Hedges LV, Rothstein HR. Basics of meta-analysis: I2 is not an absolute measure of heterogeneity. *Research Synthesis Methods* 2017; **8**(1): 5-18.

12. Peters JL, Sutton AJ, Jones DR, Abrams KR, Rushton L. Comparison of Two Methods to Detect Publication Bias in Meta-analysis. *JAMA* 2006; **295**(6): 676-80.

13. Sedgwick P. Meta-analysis: testing for reporting bias. *BMJ: British Medical Journal* 2015; **350**: g7857.

14. Hansen C, Steinmetz H, Block J. How to conduct a meta-analysis in eight steps: a practical guide. *Management Review Quarterly* 2022; **72**(1): 1-19.

15. Andersson EM, Ögren M, Molnár P, Segersson D, Rosengren A, Stockfelt L. Road traffic noise, air pollution and cardiovascular events in a Swedish cohort. *Environ Res* 2020; **185**: 109446.

16. Babisch W, Ising H, Kruppa B, Wiens D. The incidence of myocardial infarction and its relation to road traffic noise—the Berlin case-control studies. *Environ Int* 1994; **20**(4): 469-74.

17. Babisch W, Ising H, Gallacher JE, Sweetnam PM, Elwood PC. Traffic noise and cardiovascular risk: the Caerphilly and Speedwell studies, third phase--10-year follow up. *Arch Environ Health* 1999; **54**(3): 210-6.

18. Babisch W, Beule B, Schust M, Kersten N, Ising H. Traffic noise and risk of myocardial infarction. *Epidemiology* 2005; **16**(1): 33-40.

19. Bai L, Shin S, Oiamo TH, Burnett RT, Weichenthal S, Jerrett M, *et al*. Exposure to Road Traffic Noise and Incidence of Acute Myocardial Infarction and Congestive Heart Failure: A Population-Based Cohort Study in Toronto, Canada. *Environ Health Perspect* 2020;**128**(8): 87001.

20. Bodin T, Björk J, Mattisson K, Bottai M, Rittner R, Gustavsson P, *et al*. Road traffic noise, air pollution and myocardial infarction: a prospective cohort study. *Int Arch Occup Environ Health* 2016; **89**(5): 793-802.

21. Carey IM, Anderson HR, Atkinson RW, Beevers S, Cook DG, Dajnak D, *et al*. Traffic pollution and the incidence of cardiorespiratory outcomes in an adult cohort in London. *Occup Environ Med* 2016; **73**(12): 849-56.

22. Cole-Hunter T, Dehlendorff C, Amini H, Mehta A, Lim YH, Jørgensen JT, *et al*. Long-term exposure to road traffic noise and stroke incidence: a Danish Nurse Cohort study. *Environ Health* 2021; **20**(1): 115.

23. Dimakopoulou K, Koutentakis K, Papageorgiou I, Kasdagli MI, Haralabidis AS, Sourtzi P, *et al*. Is aircraft noise exposure associated with cardiovascular disease and hypertension? Results from a cohort study in Athens, Greece. *Occup Environ Med* 2017; **74**(11): 830-7.

24. Grady ST, Hart JE, Laden F, Roscoe C, Nguyen DD, Nelson EJ, *et al*. Associations between long-term aircraft noise exposure, cardiovascular disease, and mortality in US cohorts of female nurses. *Environ Epidemiol* 2023; **7**(4).

25. Hao G, Zuo L, Weng X, Fei Q, Zhang Z, Chen L, *et al*. Associations of road traffic noise with cardiovascular diseases and mortality: Longitudinal results from UK Biobank and meta-analysis. *Environ Res* 2022; **212**(Pt A): 113129.

26. Lim YH, Jørgensen JT, So R, Cramer J, Amini H, Mehta A, *et al*. Long-term exposure to road traffic noise and incident myocardial infarction: A Danish Nurse Cohort study. *Environ Epidemiol* 2021; **5**(3): e148.

27. Magnoni P, Murtas R, Russo AG. Residential exposure to traffic-borne pollution as a risk factor for acute cardiocerebrovascular events: a population-based retrospective cohort study in a highly urbanized area. *Int J Epidemiol* 2021; **50**(4): 1160-71.

28. Poulsen AH, Sørensen M, Hvidtfeldt UA, Christensen JH, Brandt J, Frohn LM, *et al*. Concomitant exposure to air pollution, green space, and noise and risk of stroke: a cohort study from Denmark. *Lancet Reg Health Eur* 2023; **31**: 100655.

29. Pyko A, Andersson N, Eriksson C, de Faire U, Lind T, Mitkovskaya N, *et al*. Long-term transportation noise exposure and incidence of ischaemic heart disease and stroke: a cohort study. *Occup Environ Med* 2019; **76**(4): 201-7.

30. Roswall N, Raaschou-Nielsen O, Ketzel M, Gammelmark A, Overvad K, Olsen A, *et al*. Long-term residential road traffic noise and NO2 exposure in relation to risk of incident myocardial infarction - A Danish cohort study. *Environ Res* 2017; **156**: 80-6.

31. Seidler A, Wagner M, Schubert M, Dröge P, Pons-Kühnemann J, Swart E, *et al*. Myocardial Infarction Risk Due to Aircraft, Road, and Rail Traffic Noise. *Dtsch Arztebl int* 2016; **113**(24): 407-14.

32. Seidler AL, Hegewald J, Schubert M, Weihofen VM, Wagner M, Dröge P, *et al*. The effect of aircraft, road, and railway traffic noise on stroke - results of a case-control study based on secondary data. *Noise Health* 2018; **20**(95): 152-61.

33. Selander J, Nilsson ME, Bluhm G, Rosenlund M, Lindqvist M, Nise G, *et al*. Long-term exposure to road traffic noise and myocardial infarction. *Epidemiology* 2009; **20**(2): 272-9.

34. Sørensen M, Andersen ZJ, Nordsborg RB, Jensen SS, Lillelund KG, Beelen R, *et al*. Road traffic noise and incident myocardial infarction: a prospective cohort study. *PLoS ONE* 2012; **7**(6): e39283.

35. Sørensen M, Lühdorf P, Ketzel M, Andersen ZJ, Tjønneland A, Overvad K, *et al*. Combined effects of road traffic noise and ambient air pollution in relation to risk for stroke? *Environ Res* 2014; **133**: 49-55.

36. Sørensen M, Poulsen AH, Hvidtfeldt UA, Münzel T, Thacher JD, Ketzel M, *et al*. Transportation noise and risk of stroke: a nationwide prospective cohort study covering Denmark. *Int J Epidemiol* 2021; **50**(4): 1147-56.

37. Thacher JD, Poulsen AH, Raaschou-Nielsen O, Hvidtfeldt UA, Brandt J, Christensen JH, *et al*. Exposure to transportation noise and risk for cardiovascular disease in a nationwide cohort study from Denmark. *Environ Res* 2022; **211**: 113106.

38. Vienneau D, Saucy A, Schäffer B, Flückiger B, Tangermann L, Stafoggia M, *et al*. Transportation noise exposure and cardiovascular mortality: 15-years of follow-up in a nationwide prospective cohort in Switzerland. *Environ Int* 2022; **158**: 106974.

39. Willich SN, Wegscheider K, Stallmann M, Keil T. Noise burden and the risk of myocardial infarction: False interpretation of results due to inadequate treatment of data: reply [6]. *Eur Heart J* 2006; **27**(5): 624.

40. Yankoty LI, Gamache P, Plante C, Goudreau S, Blais C, Perron S, *et al*. Long-term residential exposure to environmental/transportation noise and the incidence of myocardial infarction. *Int J Hyg Environ Health* 2021; **232**: 113666.

41. Wells GA, Shea B, O’Connell D, Peterson J, Welch V, Losos M, *et al*. The Newcastle-Ottawa Scale (NOS) for assessing the quality if nonrandomised studies in meta-analyses. 2012
